# A Novel Marine Pathogen Isolated from Wild Cunners (*Tautogolabrus adspersus*): Comparative Genomics and Transcriptome Profiling of *Pseudomonas* sp. Strain J380

**DOI:** 10.3390/microorganisms9040812

**Published:** 2021-04-12

**Authors:** Navaneethaiyer Umasuthan, Katherinne Valderrama, Ignacio Vasquez, Cristopher Segovia, Ahmed Hossain, Trung Cao, Hajarooba Gnanagobal, Jennifer Monk, Danny Boyce, Javier Santander

**Affiliations:** 1 Marine Microbial Pathogenesis and Vaccinology Laboratory, Department of Ocean Sciences, Memorial University of Newfoundland, St. John’s, NL A1C 5S7, Canada; navaumasuthan@gmail.com (N.U.); kvalderrama@mun.ca (K.V.); ivasquezsoli@mun.ca (I.V.); cwsegovia@mun.ca (C.S.); ahossain@mun.ca (A.H.); ttcao@mun.ca (T.C.); hgnanagobal@mun.ca (H.G.); 2 Dr. Joe Brown Aquatic Research Building (JBARB), Department of Ocean Sciences, Memorial University of Newfoundland, Logy Bay, NL A1C 5S7, Canada; jmonk@mun.ca (J.M.); dboyce@mun.ca (D.B.)

**Keywords:** cleaner fish, *Salmo salar*, bacterial infection, comparative genomics, transcriptomics, iron homeostasis

## Abstract

Cunner (*Tautogolabrus adspersus*) is a cleaner fish being considered for utilized in the North Atlantic salmon (*Salmo salar*) aquaculture industry to biocontrol sea lice infestations. However, bacterial diseases due to natural infections in wild cunners have yet to be described. This study reports the isolation of *Pseudomonas* sp. J380 from infected wild cunners and its phenotypic, genomic, and transcriptomic characterization. This Gram-negative motile rod-shaped bacterium showed a mesophilic (4–28 °C) and halotolerant growth. Under iron-limited conditions, *Pseudomonas* sp. J380 produced pyoverdine-type fluorescent siderophore. Koch’s postulates were verified in wild cunners by intraperitoneally (i.p.) injecting *Pseudomonas* sp. J380 at 4 × 10^3^, 4 × 10^5^, and 4 × 10^7^ colony forming units (CFU)/dose. Host-range and comparative virulence were also investigated in lumpfish and Atlantic salmon i.p. injected with ~10^6^ CFU/dose. Lumpfish were more susceptible compared to cunners, and Atlantic salmon was resistant to *Pseudomonas* sp. J380 infection. Cunner tissues were heavily colonized by *Pseudomonas* sp. J380 compared to lumpfish and Atlantic salmon suggesting that it might be an opportunistic pathogen in cunners. The genome of *Pseudomonas* sp. J380 was 6.26 megabases (Mb) with a guanine-cytosine (GC) content of 59.7%. Biochemical profiles, as well as comparative and phylogenomic analyses, suggested that *Pseudomonas* sp. J380 belongs to the *P. fluorescens* species complex. Transcriptome profiling under iron-limited vs. iron-enriched conditions identified 1159 differentially expressed genes (DEGs). Cellular metabolic processes, such as ribosomal and energy production, and protein synthesis, were impeded by iron limitation. In contrast, genes involved in environmental adaptation mechanisms including two-component systems, histidine catabolism, and redox balance were transcriptionally up-regulated. Furthermore, iron limitation triggered the differential expression of genes encoding proteins associated with iron homeostasis. As the first report on a bacterial infection in cunners, the current study provides an overview of a new marine pathogen, *Pseudomonas* sp. J380.

## 1. Introduction

The ectoparasitic infestation by sea louse (e.g., *Lepeophtheirus salmonis*) is the most serious threat to both wild and cage-cultured salmonids (e.g., *Salmo salar*) in the Northern hemisphere [1]. During the infestation, sea lice feed on salmonid tissues leading to skin erosion, osmoregulatory failure, immunosuppression, increased disease susceptibility and, ultimately, death [1,2]. Sea lice escalate the economic burden on salmon farmers both by causing mortalities and demanding expensive control regimes [3].

Over the years, the Atlantic salmon industry has used several methods to combat sea lice infestation. Chemotherapeutic treatment was the dominant sea lice control method for decades until the recent emergence of resistance in sea lice to several active components of drugs [4] and increasing negative public opinion about the impacts of anti-lice drugs on ecological equilibrium. Mechanical abrasion and thermal treatment are routinely used as physical delousing methods, but have adverse effects on salmon health and welfare [5]. Integrated pest management (IPM) was then introduced to the salmon industry, in which multiple non-medicinal methods are being utilized in different combinations (e.g., physical barriers—curtains and skirts, anti-lice diets, laser, water jets, and ultrasound) [6].

Biological pest control by using cleaner fishes is another commonly used lice-controlling strategy in North Atlantic salmon farms [7]. Cleaner fish are considered as ‘green alternatives’ and the potential solution to lice infestation from both economic and ecological points of view. The mutually beneficial cleaner fish—salmonid association reduces the parasite burden on salmonids while providing a food source to cleaner fish. In salmonid aquaculture, different wrasse (e.g., rock cook (*Centrolabrus exoletus*), goldsinny (*Ctenolabrus rupestris*), cuckoo (*Labrus mixtus*), ballan (*Labrus bergylta*), and corkwing (*Symphodus melops*) and lumpfish (*Cyclopterus lumpus*) species are used [8,9,10]. Wrasses and lumpfish possess contrasting season-dependent feeding behaviors. Wrasses tend to consume more parasites than lumpfish, but they reduce their activity during winter and eventually enter into hypometabolic winter dormancy (torpor) state at water temperatures below 5 °C [11]. However, as a cold-water cleaner fish, lumpfish can effectively delouse at or even below, 5 °C. Therefore, the usage of multiple cleaner fish species in combination (e.g., cohabiting wrasse and lumpfish) might be advantageous, since they are efficient during the spring–summer period and autumn–winter period, respectively [10,12].

The potential of cunner (*Tautogolabrus adspersus*), a native Atlantic Canadian species [13], as a delousing fish was first studied by MacKinnon in the 1990s [14]. A more recent study also described the cleaning behavior and delousing efficiency of cunners using a *S. salar*—*L. salmonis* model [15]. The increasing interest in the commercialization of cleaner fish in Atlantic Canada may lead to the consideration of cultured cunners for hatchery production in the future [16]. However, infectious diseases in cunners are not well described [17].

*Pseudomonas* spp. exist in a diverse range of environments including marine habitats. Some pathogenic species, such as *P. fluorescens* [18], *P. putida* [19], and *P. plecoglossicida* [20], have been reported to cause diseases in rainbow trout (*Oncorhynchus mykiss*), ayu (*Plecoglossus altivelis*), and tilapia (*Oreochromis niloticus*) [21].

Iron is indispensable for several bacterial processes including DNA synthesis, enzyme and redox catalysis, electron transport, and respiration, and is considered a central determinant for growth, survival, and pathogenesis. Bacterial habitats are commonly limited in iron availability and iron withholding is a principal defense strategy imposed by the host to prevent microbial outgrowth. In order to successfully establish an infection, pathogens have evolved many sophisticated evading mechanisms (e.g., siderophore iron-sequesters) for iron piracy from their hosts [22,23]. On the other hand, due to the extreme toxicity resulting from iron overload, bacteria employ a variety of mechanisms to regulate intracellular iron concentrations by coordinating complex transcriptional regulatory schemes to control multiple aspects of iron homeostasis. The uptake, transport, storage, and mobilization of iron are controlled in an iron-dependent manner [22,23].

In this study, we isolated and studied a bacterial pathogen (named *Pseudomonas* sp. strain J380) that caused skin lesions and ulcers in wild-caught cunners from the coast of Eastern Newfoundland (Canada). Koch’s postulates and infection kinetics were studied in cunners under controlled conditions. Then, host-range was evaluated in lumpfish and Atlantic salmon. *Pseudomonas* sp. J380 was determined to infect both cleaner fish species, but not Atlantic salmon. This new species was then characterized at phenotypic, genomic, and transcriptomic levels. Comparative genomics and phylogenetic inference indicated that *Pseudomonas* sp. J380 is a new strain and closely related to the *P. fluorescens* species complex. Finally, RNA-Seq transcriptome profiling under iron-enriched vs. iron-limited conditions revealed a modulated expression of canonical and unique genes potentially linked to metabolism, environmental adaptation mechanisms, and iron homeostasis. Collectively, our findings provide insights into the biology of a novel marine pathogen, *Pseudomonas* sp. J380.

## 2. Materials and Methods

### 2.1. Pseudomonas sp. Strain J380 Isolation

*Pseudomonas* sp. strain J380 was isolated from the head kidney, liver, and spleen of infected wild cunners captured at Conception Bay, Newfoundland and Labrador (NL), Canada (Figure S1A,B). Fish with skin lesions (Figure S1C,D) were netted and euthanized with an overdose of MS222 (400 mg/L) (Syndel Laboratories, BC, Canada). Samples were dissected and placed into sterile homogenizer bags (Nasco Whirl-Pak^®^, Madison, WI, USA). The infected tissues were weighed and homogenized in phosphate-buffered saline (PBS; 136 mM NaCl, 2.7 mM KCl, 10.1 mM Na_2_HPO_4_, 1.5 mM KH_2_PO_4_ (pH 7.2)) up to a final volume of 1 mL. Homogenized and serially diluted tissue suspension (100 µL) was plated onto Trypticase Soy Agar (TSA) plates supplemented with 2% NaCl and incubated at 15 °C for 48 h. Isolated colonies were selected and purified for further analysis. Bacterial stocks were preserved at −80 °C in 10% glycerol and 1% peptone solution. Three selected colonies were phenotypically characterized and subjected to 16S sequencing using universal primers, 27F (5’-AGAGTTTGATYMTGGCTCAG-3’) and 1492R (5′-TACGGYTACCTTGTTACGACTT-3′) [24] at Core Science Facility, Memorial University of Newfoundland (MUN).

### 2.2. Bacterial Growth Conditions

Single colonies of *Pseudomonas* sp. J380 were grown routinely in 3 mL of Trypticase Soy Broth (TSB, Difco, Franklin Lakes, NJ, USA) at 15 °C in a 16 mm diameter glass tube and placed in a roller for 24 h. To conduct the assays under iron-enriched and -limited conditions, TSB was supplemented with 100 µM FeCl_3_ or 100 µM 2,2’-dipyridyl (DIP), respectively. Siderophore synthesis was detected on Chrome Azurol S (CAS) agar plates [25] using bacterial cells harvested at the mid-log phase at an optical density (OD; at 600 nm) of ~0.7 (~1 × 10^8^ CFU/mL).

### 2.3. Biochemical, Enzymatic, and Physiological Characterization

The biochemical and enzymatic profiles of *Pseudomonas* sp. J380 were characterized using standard strips systems, including API20E, API20NE, and APY ZYM (BioMerieux, Marcy-l’Etoile, France) according to the manufacturer’s instructions. Following the incubation of strips with *Pseudomonas* sp. J380 at 15 °C for 48 h, the results were analyzed using APIWEB (BioMerieux). The growth of *Pseudomonas* sp. J380 was evaluated at different temperatures (4 °C, 15 °C, 28 °C, and 37 °C) and NaCl concentrations (0%, 1%, and 2%). Motility, siderophore synthesis, hemolysis activity, catalase activity, and oxidase activity were evaluated using standard methods [26]. The antibiogram of *Pseudomonas* sp. J380 was determined for tetracycline (TET; 30 µg), oxytetracycline (OTC; 30 µg), ampicillin (AMP; 10 µg), chloramphenicol (CHL; 30 µg), trimethoprim-sulfamethoxazole (SXT; 25 µg), cefotaxime (CXT; 30 µg), and oxolinic acid (OXA; 2 µg) using standard methods [27].

### 2.4. Siderophores Synthesis

The secretion of siderophores was tested using CAS plates by previously described assays [28,29]. *Pseudomonas* sp. J380 was grown under previously described conditions. Briefly, bacterial cells grown in TSB were harvested at the mid-log phase. Thirty microliters of bacterial inoculum were added to 3 mL TSB supplemented with 100 µM of FeCl_3_ or 100 µM of DIP and grown at 15 °C for 24 h with aeration. As a control, *Vibrio anguillarum* J360 was grown under the same conditions but in TSB supplemented with 2% NaCl [30]. Following the incubation period, the cells were harvested (6000 rpm, 10 min), washed twice with PBS, and resuspended in 50 µL of PBS. Ten microliters of the concentrated bacterial pellet were inoculated onto CAS agar plates and incubated at 15 °C for 48–72 h. Following the incubation, the secreted siderophores were visualized as a yellow-orange halo around the bacterial colony. The plates were also observed using a UV trans-illuminator. To photo-document the fluorescent siderophores, bacteria grown under previously described conditions were washed and resuspended in 1 mL of PBS and stained with 4’,6-diamidino-2-phenylindole (DAPI; Thermo Fisher, USA) for 30 min in darkness. After washing cells with PBS three times (6000 rpm, 10 min), cells were visualized using DAPI (461 nm) and EtBr (358 nm) filters through a Nikon AR1 laser scanning confocal microscope.

### 2.5. Fish Capture and Holding

Fish were captured or produced and maintained in tanks within the Dr. Joe Brown Aquatic Research Building (JBARB) at the Department of Ocean Sciences (DOS), MUN, under animal protocols #18-01-JS, #18-03-JS (21 Jan 2018), and biohazard L-01. All the protocols were reviewed and approved by the Institutional Animal Care Committee (https://www.mun.ca/research/about/acs/acc/) following the Canadian Council of Animal Care guidelines (https://www.ccac.ca/).

Cunner fish (*Tautogolabrus adpersus*) captured in Conception Bay, NL, Canada (Figure S1), were transferred to JBARB for 4 weeks of quarantine. Upon arrival, fish were acclimated to ~8–10 °C in 500 L tanks supplied with oxygen saturation of 95–110%, UV-treated, and filtered flow-through seawater, with 12–12 photoperiod and illuminance of 10–15 lux. The animals were fed with chopped capelin (*Mallotus villosus*) two times per week *ad libitum*. Lumpfish were acclimated to ~8–10 °C in 500 L tanks supplied with oxygen saturation of 95–110%, UV-treated, and filtered flow-through seawater, and 12–12 photoperiod with an illuminance of 30 lux. Biomass was maintained at 6.6 kg/m^3^. The fish were fed a commercial diet (Skretting—Europa, BC, Canada) daily at a rate of 0.5% of body weight per day using automated feeders. Farmed Atlantic salmon were held under optimal conditions in 3800 L tanks as described previously [31], with some modifications (at 10 °C, and a 12/12 photoperiod with an illuminance of 40–60 lux). The fish were fed three days per week at a level of 1% body weight per day using a commercial dry pellet (Skretting–Europa).

### 2.6. Infection Assays

Wild cunners (~150 ± 50 g)**,** cultured lumpfish (~140 ± 20 g), and cultured Atlantic salmon (~800 ± 100 g) were transferred from JBARB to AQ3 biocontainment at Cold-Ocean Deep-Sea Research Facility (CDRF) at DOS, MUN, for infection assays and acclimated for 2 weeks under the above-described conditions. Cunners and lumpfish were separated into 500 L tanks containing 60 fish per tank and Atlantic salmon were separated into six tanks containing 15 fish each (two tanks/dose; 30 fish/dose). The infection procedures were conducted according to established protocols [30,32,33]. Briefly, fish were anesthetized with 50 mg/L MS222 per liter of seawater and intraperitoneally (i.p.) injected with 100 µL of inoculum. Cunners were injected with three doses of 4 × 10^3^ (low), 4 × 10^5^ (medium), and 4 × 10^7^ (high) CFU/dose, and a control group (*n* = 60) was mock injected with PBS. In an independent experiment, lumpfish and Atlantic salmon were injected with 1 × 10^6^ and 2.2 × 10^6^ CFU/dose of *Pseudomonas* sp. J380, respectively. Mortality was monitored daily until ~30 days post-injection (dpi). Samples of liver, spleen, and head kidney were taken from moribund fish to re-isolate the pathogen.

### 2.7. Tissue Sampling and Analysis

After infection, samples of internal organs (liver, spleen, head kidney, brain, and blood), were taken from a minimum of 5 fish euthanized with an overdose of MS222 (400 mg/L) in every sampling. Tissue samples were aseptically removed at 7, 14, 21, and 35 dpi for cunners; 7, 14, 21, and 28 dpi for lumpfish; and 7 and 14 for Atlantic salmon, based on the progression of the disease. The samples were placed into sterile homogenizer bags. Subsequently, they were weighed and homogenized in PBS in a final volume of 1 mL (weight: volume), serially diluted in PBS (1:10), and plated on TSA plates before incubating at 15 °C for 3–4 days. The following formula was used to determine the CFU of *Pseudomonas* sp. J380 per g of tissue [34]:

(1) $$\mathrm{CFU}\;\times {\;\mathrm{gram}}^{-1}\;=\;\frac{CFU\;\times \;(1/Dilution\;Factor)\;\times 10}{Tissue\;weight\;\left(grams\right)}$$

### 2.8. DNA Extraction and Sequencing

*Pseudomonas* sp. J380 genomic DNA was extracted from cultures grown to mid-logarithmic phase as described in Section 2.2. The Wizard DNA extraction high molecular weight kit (Promega, Madison, WI, USA) was used to extract and purify the genomic DNA. Integrity and purity of the DNA were evaluated by gel electrophoresis (0.8% agarose) and spectrophotometry (Genova-Nano spectrophotometer, Jenway, UK). Library preparations and sequencing were conducted by Genome Quebec (ON, Canada) using PacBio RS II and Miseq Illumina sequencers.

### 2.9. Genome Assembly, Annotation, and Mapping

Celera Assembler (August 2013; version v7.0) at Genome Quebec was used to assemble the PacBio reads. Rapid Annotation Subsystem Technology pipeline (RAST) was used for the annotation [35]. The *Pseudomonas* sp. J380 chromosome was submitted to the National Center for Biotechnology Information (NCBI) and re-annotated using the NCBI Prokaryotic Genome Annotation Pipeline (PGAP; 4.10).

To detect small plasmids, the raw Illumina reads were trimmed using CLC Genomics Workbench v20.0 (CLCGWB; Qiagen, Hilden, Germany) and examined for quality using FastQC version 0.11.9 (Babraham Institute, Cambridge, UK) [36]. High-quality Illumina reads were assembled using *de novo* tool (CLCGWB) and aligned to the reference *Pseudomonas* sp. J380 chromosome using the genome finishing module tools with default parameters. Illumina sequences that did not align with the chromosome were analyzed and annotated. *Pseudomonas* sp. J380 whole-genome illustration was developed by using CGview Server [37].

### 2.10. Multi-Locus Sequence Analysis (MLSA) Using Housekeeping Genes

To infer the phylogenetic history of *Pseudomonas* sp. J380, several reference genes from different pseudomonads with complete genomes were considered. These genes included 16S ribosomal RNA subunit (*rrn*), cell-division protein (*ftsZ*), glyceraldehyde-3-phosphate dehydrogenase (*gapA*), gyrase beta subunit (*gyrB*), rod shape-determining protein (*mreB*), uridine monophosphate (UMP) kinase or uridylate kinase (*pyrH*), recombinase A (*recA*), RNA polymerase alpha subunit (*rpoA*), and topoisomerase I (*topA*) and were used in MLSA. The gene loci and accession numbers for each species are listed in Table S1. Sequences were aligned using CLCGWB. Concatenation of locus sequence was made using Sequence Matrix software v1.7.8 [38]. The phylogenetic history was estimated using the neighbour-joining (NJ) method [39] with a bootstrap consensus of 1000 replicates using MEGA X.

### 2.11. Whole Genome Comparison and Evolutionary Analysis

The genomes used in this study are listed in Table S2, and the analyses were conducted using the whole-genome analysis tool (CLCGWB) as described earlier [30]. To determine the average nucleotide identity (ANI), genomes were aligned with default parameters (min. initial seed length = 15; allow mismatches = yes; min. alignment block = 100). A correlation matrix was generated with default parameters (Euclidean distance method and complete cluster linkages). The phylogenetic history was determined using NJ method [39] with a bootstrap consensus of 1000 replicates using CLCGWB. *Aeromonas salmonicida* strain J223 (NZ_CP048223) was used as an outgroup. The analysis was repeated using MEGA X using the same parameters [40]. Whole-genome dot plots between closely related strains were also constructed to visualize and further analyze genomic differences.

### 2.12. Bacterial Growth under Iron-Enriched and -Limited Conditions, and Total RNA Extraction

Triplicate cultures of 50 mL of *Pseudomonas* sp. J380 grown under iron-enriched and iron-limited conditions at 15 °C with aeration (180 rpm) to mid-log phase were subjected for RNA extraction according to established protocols [31,41]. Briefly, cells were harvested (6000 rpm, 10 min, 4 °C) and washed twice with PBS. The cell pellet was used for total RNA extraction using MirVana following the manufacturer’s instructions (Invitrogen, Waltham, MA, USA). To degrade any residual genomic DNA, the RNA samples were treated with 2 U of DNase (TURBO DNA-free™ Kit, Invitrogen) and incubated at 37 °C for 30 min. Then, 2.5 μL of DNase Inactivation Reagent was added, and further incubated for 5 min at room temperature. The supernatant containing clean RNA was harvested by centrifuging samples at 10,000× *g* for 1.5 min. RNA samples were quantified and evaluated for purity (A260/280 and A260/230 ratios) using a GenovaNano-spectrophotometer (Jenway, UK). RNA integrity was evaluated by agarose gel electrophoresis. The A260/280 and A260/230 ratios of purified RNA samples were 1.9–2.1 and 1.9–2.2, respectively.

### 2.13. Library Preparation and RNA-Sequencing

For each condition, there were three biological replicates (total = 6 samples). Library preparation and sequencing were done commercially at Genome Quebec. Briefly, RNA quality was evaluated using a NanoDrop spectrophotometer (Thermo Scientific) and a Bioanalyzer 2100 (Agilent; Figure S2). Libraries were generated using the NEBNext^®^ Multiplex Oligos for Illumina^®^ (Dual Index Primers Set 1; Adapter 1: 3’-AGATCGGAAGAGCACACGTCTGAACTCCAGTCAC-5’; Adapter 2: 3’-AGATCGGAAGAGCGTCGTGTAGGGAAAGAGTGT-5’) and RNA depleted (1 ng; 5S, 16S, 23S) using NEBNext^®^ rRNA Depletion Kit (Bacteria) according to the manufacturer’s instructions. Sequencing runs were performed on NovaSeq 6000 Sequencer (Illumina) using a NovaSeq 6000 S4 100 bp PE flow cell.

### 2.14. RNA-Seq Data Analysis

The RNA-Seq data is available at the NCBI database under the accession number PRJNA717273. Obtained paired-end raw reads were mate-paired and filtered to remove low-quality reads using CLCGWB with default parameters (mate-paired read information, minimum distance = 1; maximum distance = 1000) (Table S3). Adapter trimming was realized by CLCGWB using the trim reads tool with default parameters (quality trimming, trim using quality scores, limit: 0.05, and trim ambiguous nucleotides, maximum number of ambiguities = 2). The number of reads and nucleotides removed are indicated in Table S3. The quality of the reads was checked using FastQC [36] before and after trimming. Trimmed high-quality reads were, then, mapped by CLCGWB against the *Pseudomonas* sp. J380 genome (NZ_CP043060.1) using the RNA-Seq analysis tool. Reads mapping and transcript counts were performed using the following settings: mismatch cost = 2, insertion and deletion costs = 3, minimum length fraction and minimum similarity fraction = 0.8, maximum number of hits for a read = 10, and strand-specific = both. Gene expression quantification and normalization of the mapped reads were performed by alignment-dependent expectation-maximization (EM) algorithm [42] based on the RESM and eXpress methods [43]. The transcript per million reads (TPM) values were, then, computed from the counts assigned to each transcript, after normalization by the trimmed mean of M-values (TMM) [44]. A global correlation analysis was performed using log_2_-transformed TPM values (x + 1) of each gene under iron-enriched and iron-limited conditions. The correlation was estimated by Pearson method. Abundance data were subsequently subjected to differential expression analyses using the CLCGWB and the differential expression tool based on a negative binomial general linear model (GLM) [45]. A standard selection of biologically significant differentially expressed genes (DEGs) was performed with cut-off values of log_2_ fold-change (FC) ≥ |1| and false discovery rate (FDR), *p* ≤ 0.05.

### 2.15. Analyses of Enriched Gene Ontology (GO) Terms and KEGG Kyoto Encyclopedia of Genes and Genomes Pathways

DEGs identified in this study under standard selection criteria were subjected to Gene Ontology (GO) and KEGG enrichment analyses by ShinyGO version 0.61 [46]. GO terms and KEGG pathways with FDR, *p* ≤ 0.05 were considered as significantly enriched, and the top 20 elements were selected to construct graphical illustration using ggplot2 version 3.3.1 [47]. A GO term-gene network analysis was performed using ClueGO plug-in [48] in Cytoscape (v3.8.2). Three GO category resources (updated on 23.03.2021) for biological process (BP), molecular function (MF), and cellular component (CC) were used.

### 2.16. In Silico Tools

*Pseudomonas* Genome DB (PGDB; https://pseudomonas.com/) and homology search by DIAMOND Blast against other pseudomonads were used to determine the identity of unknown genes. The genes encoding the virulence factors were surveyed by homology search at the virulence factor database (VFDB) [49] using other *P. fluorescens* strains (i.e., pf-5, pf0-1, and SBW25) as references. In addition, the prediction of Fur box sequences in potential target DEGs associated with iron homeostasis was carried out by regulon analysis tool at the Virtual Footprint suite [50] using *P. aeruginosa* (PAO1) as the reference.

### 2.17. Statistical Analysis

One-way ANOVA followed by a Tukey’s post hoc test was used to determine significant differences (*p* < 0.05) in percent survival. All statistical analyses were performed using GraphPad Prism 8 (GraphPad Software, San Diego, CA, USA).

## 3. Results

### 3.1. Bacterial Isolation

Wild cunners collected during the spring months (April–June) from different locations in Newfoundland have often been found to exhibit a characteristic skin ulcer. Among the wild cunners collected along the Newfoundland seashore in Conception Bay (spring, 2017), several individuals presented with this skin ulcer (Figure S1). In order to identify the etiological agent associated with this disease, internal organs, including the liver, spleen, and head kidney were sampled from fish showing visible skin wounds. Tissue homogenates were separately plated for microbial isolation onto TSA (+2% NaCl). Colonies developed from all three tissues appeared morphologically identical and exhibited the same characteristics in multiple aspects, including colony morphology, Gram-stain, antibiogram, biochemical profile, and 16S sequencing (Table 1). This suggested that the bacterium causing lesions/ulcers in cunners is potentially a single strain capable of colonizing multiple internal organs, and received the specific strain identifier of ‘J380′.

### 3.2. Physiological Characteristics of Pseudomonas sp. J380

Morphological, biochemical, and enzymatic characteristics of *Pseudomonas* sp. J380 are summarized in Table 1. The bacteriological characterization indicated that *Pseudomonas* sp. J380 is a Gram-negative motile non-capsulated rod-shaped bacterium, which is oxidase-positive and resistant to vibriostatic agent O-129, and demonstrated mesophilic and halotolerant growth. The API20NE test (profile code 0347554) suggested *P. fluorescens* as the significant taxa with 99.4% confidence. API ZYM test showed that *Pseudomonas* sp. J380 synthesizes several enzymes including catalase, urease, alkaline phosphatase, acid phosphatase, esterase, esterase lipase, lipase, and some amino acid modifying enzymes, such as arginine dihydrolase, leucine arylamidase, valine arylamidase, and cystine arylamidase. *Pseudomonas* sp. J380 was able to assimilate glucose, mannose, and maltose (Table 1). The antibiogram analysis further showed that *Pseudomonas* sp. J380 was resistant to SXT, CTX, AMP, and CHL, whereas, it was susceptible to OXA, OTC, and TET (Table 1).

In order to examine the siderophore synthesizing property, *Pseudomonas* sp. J380 and *V. anguillarum* J360 (control; [30]) were grown under iron-enriched and iron-limited conditions. Growth studies showed that the iron-limited conditions retarded the growth of both species (Figure S3A–C). However, *Pseudomonas* sp. J380 was able to grow relatively better under iron-limited conditions when compared to *V. anguillarum* J360 (Figure S3B,C). Hemolysis assay on blood agar plates showed that *Pseudomonas* sp. J380 and *V. anguillarum* J360 display β-hemolysis (only at 15 °C) and α-hemolysis (at 28 °C; [30]), respectively (Figure S3D).

**Figure 1:**
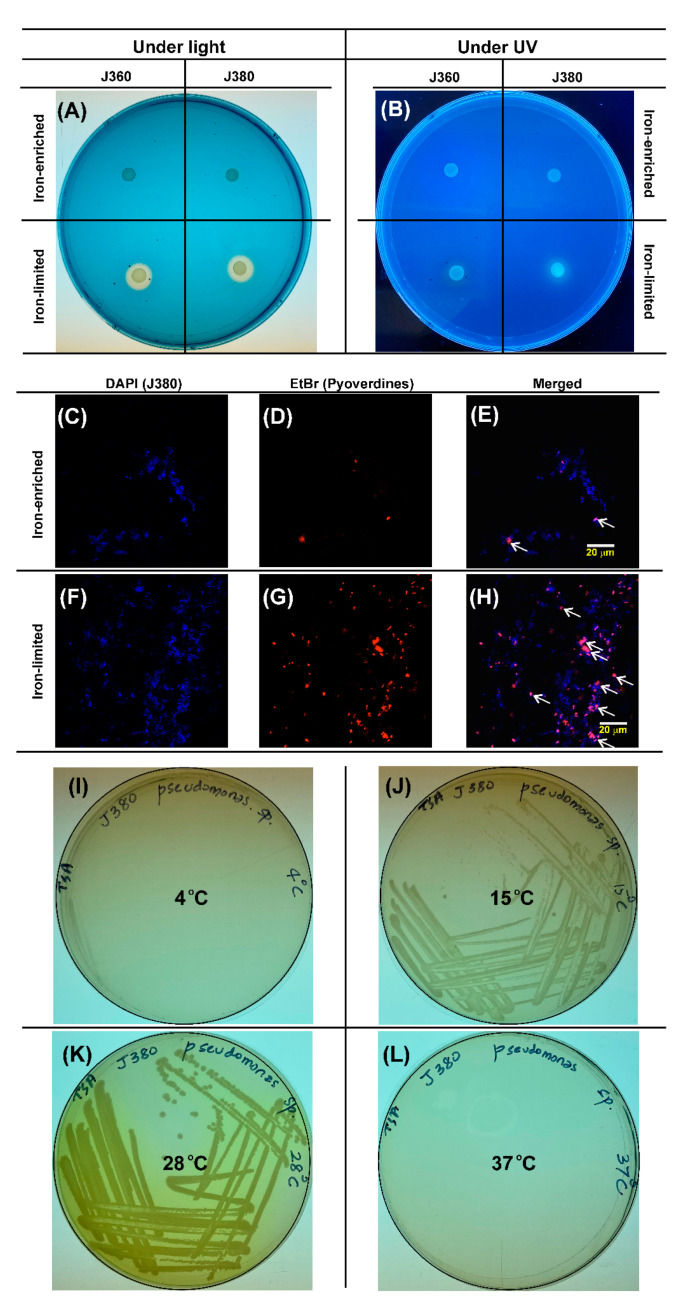
*Pseudomonas* sp. strain J380 produces fluorescent-active iron-binding siderophores (pyoverdine) under iron-limited growth conditions. *V. anguillarum* J360 represents the control. Bacteria were grown at 15 °C in trypticase soy broth (TSB) supplemented either with 100 µM FeCl_3_ or 100 µM 2,2’-dipyridyl (DIP) to provide iron-enriched and iron-limited conditions, respectively. (**A**,**B**) Blue agar CAS plate assay for two bacterial strains grown under iron-enriched and –limited conditions visualized under (**A**) normal light and (**B**) UV. Pale yellow halos around spotted cultures indicate siderophore synthesis and fluorescent halo under UV indicates pyoverdine production. (**C**–**H**) Confocal microscopic visualization of *Pseudomonas* sp. J380 labeled with DAPI. Pyoverdine secretion was evident under the EtBr filter and indicated by white arrows in merged pictograms. J380, *Pseudomonas* sp. strain J380; J360, *V. anguillarum* J360; EtBr, ethidium bromide. (**I**–**L**) Analysis of *Pseudomonas* sp. J380 growth under different temperatures. *Pseudomonas* sp. J380 was incubated onto TSA under four different temperatures for 24 h. (**I**) 4 °C (slight growth was observed), (**J**) 15 °C, (**K**) 28°C (synthesis of pyoverdine was observed; yellow/fluorescent colonies), and (**L**) 37 °C (even after 7 days of incubation, no bacterial colonies were observed).

Parallelly-grown cultures were then used in CAS plate assay. Under iron-enriched conditions, there was no siderophore production by both bacteria. In contrast, under iron-limited conditions, both *Pseudomonas* sp. J380 and *V. anguillarum* J360 synthesized siderophores and produced yellow halos with almost similar diameters in CAS plates (Figure 1A). Visualization of CAS plate under UV light revealed that the siderophores synthesized by *Pseudomonas* sp. J380, but not *V. anguillarum* J360, are fluorescent (Figure 1B), and further suggested that *Pseudomonas* sp. J380 produces pyoverdine-type siderophores under iron-limited conditions. *Pseudomonas* sp. J380 cells grown under iron-enriched and iron-limited conditions were stained with DAPI and observed under confocal microscopy. Abundant fluorescent pyoverdine was observed under iron-limited conditions (Figure 1F–H) in contrast to iron-enriched conditions (Figure 1C–E), confirming our previous observation on CAS plates under UV light (Figure 1B).

### 3.3. Determination of Koch Postulates and Pseudomonas sp. J380’s Host-Range in Lumpfish, and Atlantic Salmon

**Figure 2:**
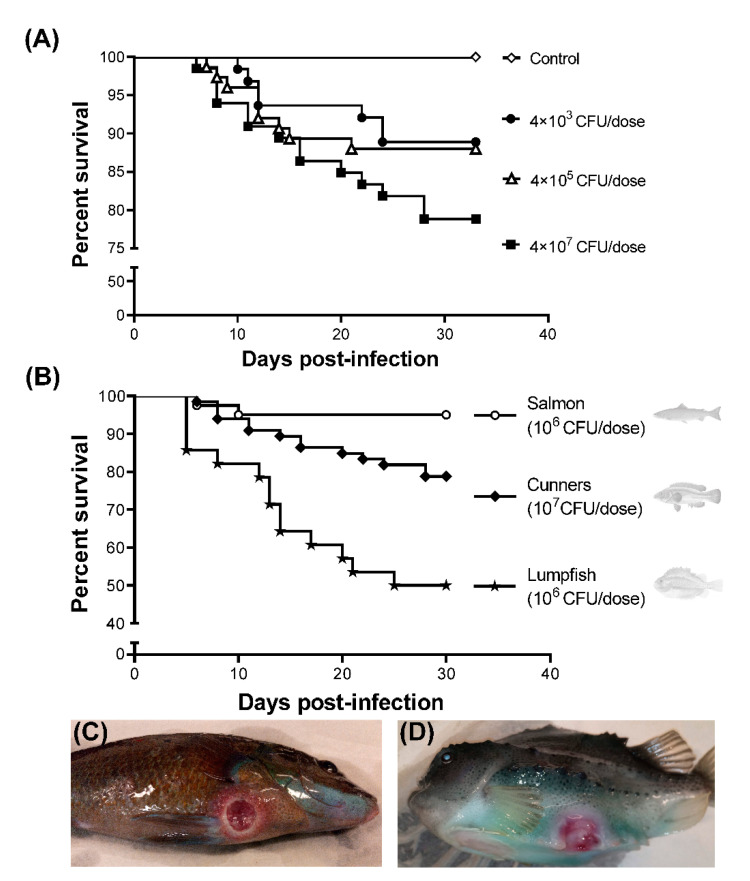
*Pseudomonas* sp. strain J380 is not an acute virulent pathogen to cunners (*Tautogolabrus adspersus*) and causes differential mortalities in hosts including, cunners, lumpfish (*Cyclopterus lumpus*), and Atlantic salmon (*Salmo salar*). (**A**) Survival assay for cunners injected with PBS (control), low (4 × 10^3^ CFU), medium (4 × 10^5^ CFU), and high (4 × 10^7^ CFU) doses of *Pseudomonas* sp. J380 per fish. Mortality was monitored for 33 dpi. (**B**) The host-range of *Pseudomonas* sp. J380 was determined by survival assays in cunners, lumpfish, and salmon challenged with 4 × 10^7^, 1 × 10^6^, and 2.2 × 10^6^ CFU/dose, and mortality was monitored for 30 dpi. (**C**,**D**) External clinical signs resulted from *Pseudomonas* sp. J380-infection in (**C**) cunner (7 dpi) and (**D**) lumpfish (5 dpi) at the advanced stage of skin ulceration. Refer to Figure S4 for additional details.

The virulence of *Pseudomonas* sp. J380 on cunners was evaluated by an i.p. injection with three different bacterial doses (low, 4 × 10^3^; medium, 4 × 10^5^; and high, 4 × 10^7^ CFU/dose) (Figure 2A). The PBS-injected control group had no clinical signs and/or mortalities throughout the experiment. Mortality began at 10 and 7 dpi for low and medium dose groups, respectively. However, these two doses caused almost similar mortalities (~12%) in cunners. At 33 dpi, ~21% of cunners in the high dose group died (Figure 2A). Overall, *Pseudomonas* sp. J380 demonstrated mild virulence in wild cunners. The highest dose used in this study did not kill all the animals and, thus, LD_50_ could not be determined (predicted to be LD_50_ > 10^8^–10^9^ CFU/dose).

The host-range of *Pseudomonas* sp. J380 was studied in lumpfish and Atlantic salmon (Figure 2B). In lumpfish infected with 1 × 10^6^ CFU/dose of *Pseudomonas* sp. J380, mortality began at 5 dpi and only 50% of lumpfish survived after 30 dpi. In Atlantic salmon infected with 2.2 × 10^6^ CFU/dose of *Pseudomonas* J380, mortality began at 6 dpi. At 30 dpi, 95% of the fish survived and displayed good health. In contrast to the deceased cunners and lumpfish (Figure 2C,D), necropsy and bacteriological analysis of the dead Atlantic salmon did not evidence bacterial infection during the infection trials. The percent survival for cunners and salmon was greater than lumpfish (Figure 2B). A significant difference was found between the percent survival of different host fish species during the *Pseudomonas* sp. J380-infection (*p* < 0.0001).

Moribund lumpfish infected with *Pseudomonas* sp. J380 revealed acute clinical signs, in contrast to cunners, in which the bacterial infection produced chronic disease characteristics (Figure 2C,D and Figure S4). In contrast, *Pseudomonas* sp. J380 caused neither apparent clinical signs nor disease in Atlantic salmon. External clinical signs in infected cunners and lumpfish included skin ulcers with hemorrhagic borders, which were superficial at the beginning of the infection, and deeper with muscle compromise at advanced stages (Figure 2C,D and Figure S4A–D). Abdominal dropsy revealed internal pathological signs in diseased fish that included splenomegaly (Figure S4E) and hemorrhagic petechiae in liver (Figure S4F,G). These features were identical characteristics that were observed in naturally infected wild cunners (Figure S1C,D).

### 3.4. Infection Kinetics of Pseudomonas sp. J380 in Different Host Tissues

**Figure 3:**
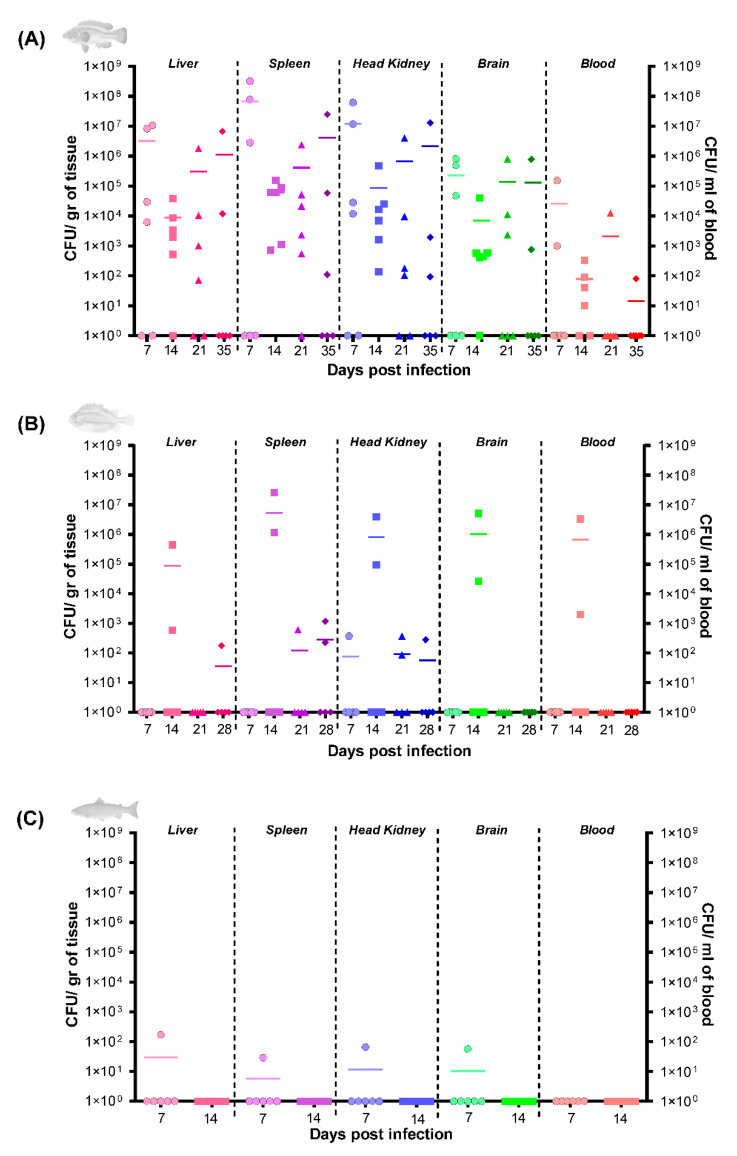
*Pseudomonas* sp. strain J380 demonstrates host-specific infection kinetics and tissue colonization in different hosts, including (**A**) Cunners (*Tautogolabrus adspersus*; *n* = 6/time point) (**B**) Lumpfish (*Cyclopterus lumpus*; *n* = 5/time point), and (**C**) Atlantic salmon (*Salmo salar*; *n* = 6/time point). For challenge details, refer to Figure 2. The symbols represent different sampling time points: circle, 7 dpi; square, 14 dpi, triangle, 21 dpi; rhomboid, 28 or 35 dpi.

The infection kinetics and tissue colonization were determined in the liver, spleen, head kidney, brain, and blood from three infected host species (Figure 3). Cunners injected with 4 × 10^7^ CFU/dose *Pseudomonas* sp. J380 were heavily colonized in all tissues sampled from the majority of the individuals, and in particular, the spleen, head kidney, and liver had >10^6^ CFU/g tissue at 7 and 35 dpi (Figure 3A). In lumpfish injected with 1 × 10^6^ CFU/dose, no bacterium was detected at 7 dpi (except in head kidney of one fish). However, two of the five lumpfish presented >10^5^ CFU/g tissue for all the tissue specimens at 14 dpi (Figure 3B). In Atlantic salmon injected with 2.2 × 10^6^ CFU/dose, a limited bacterial load was detected in tissues other than blood at 7 dpi (<10^2^ CFU/g tissue), but not at 14 dpi (Figure 3C). In summary, *Pseudomonas* sp. J380 was able to invade and colonize cunners’ tissues. Nevertheless, the degree of invasion and colonization was low in lumpfish and minimal in Atlantic salmon.

### 3.5. Genome of Pseudomonas sp. J380

**Figure 4:**
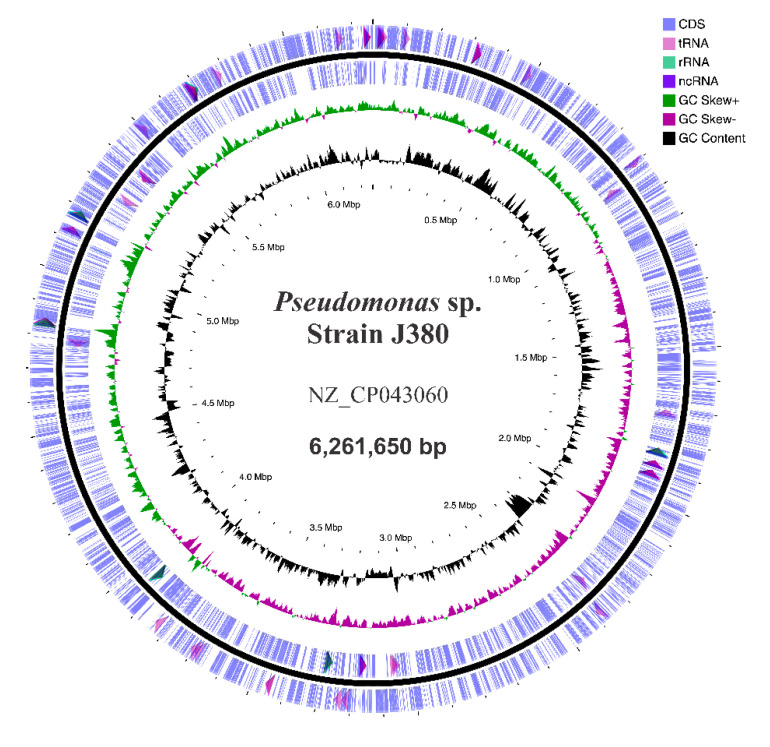
Genome map of *Pseudomonas* sp. strain J380. The genome map was generated using CGView Server. Different elements or features of the chromosome are illustrated by distinct colors as described in the legend.

Genomic DNA of *Pseudomonas* sp. J380 was sequenced using PacBio RS II and MiSeq (Illumina) platforms and assembled. The genome of *Pseudomonas* sp. J380 has been deposited at NCBI under the accession number NZ_CP043060, BioProject (acc. no. PRJNA561239), and BioSample (acc. no. SAMN12612376). The genome of *Pseudomonas* sp. J380 had a single chromosome with a total length of 6,261,650 bp with a GC content of 59.7% (Figure 4, Table 2). No plasmids were detected by using gel electrophoresis or MiSeq data. PGAP at NCBI determined a total of 5734 genes, including 5568 genes with CDSs, six complete rRNA operons, four ncRNAs, and 74 pseudogenes (Table 2).

A homology search at VFDB identified 73 putative genes encoding virulence factors in the *Pseudomonas* sp. J380 genome (Table S4), and were found to be associated with flagella, lipopolysaccharides, phenazines and alginate biosynthesis, phospholipases, and pyoverdines.

### 3.6. Multi-Locus Sequence Analysis (MLSA) of Pseudomonas sp. J380

In order to understand the phylogenic history of *Pseudomonas* sp. J380, an MLSA was conducted using eight different canonical genes from different bacterial species (Table S1). *Pseudomonas* sp. J380 demonstrated a closer relationship with two other *P. fluorescens* strains (i.e., L321 and FW300-N2C) with 100% bootstrap support (Figure S5).

### 3.7. Whole Genome Alignment and Phylogenomic Analyses

**Figure 5:**
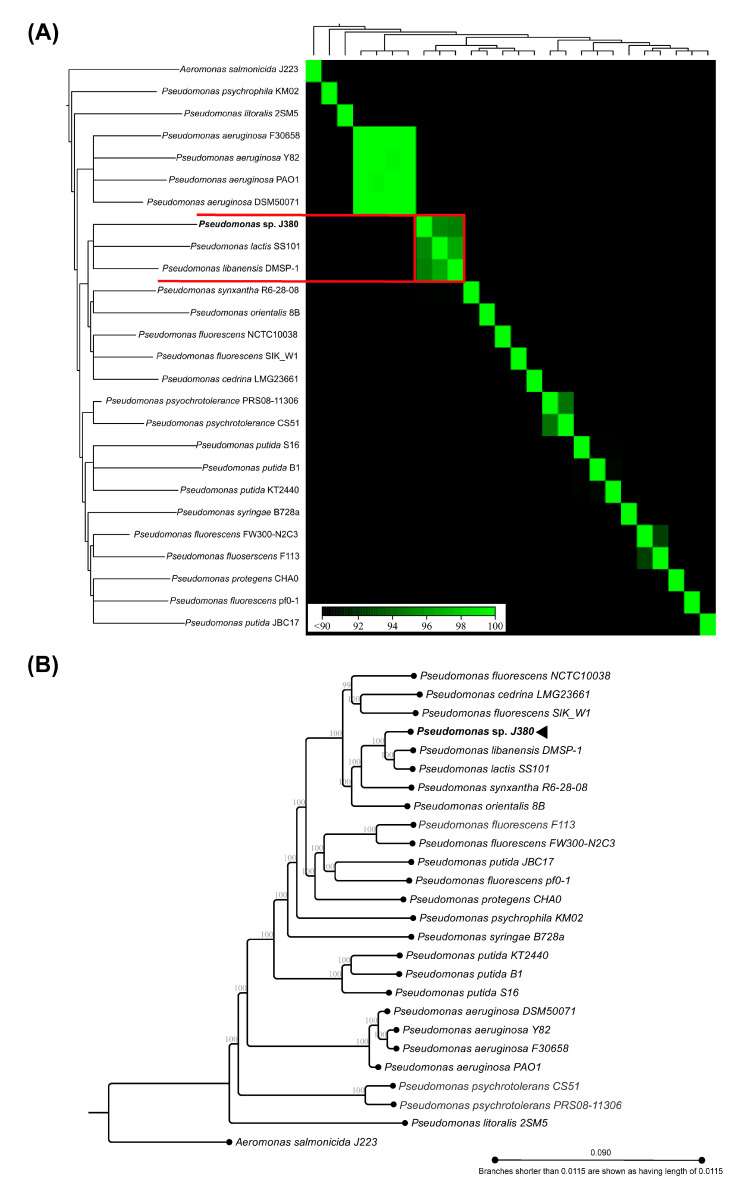
Comparative and phylogenomic analyses revealing clustering of *Pseudomonas* sp. J380 with members of *P. fluorescens* species complex. (**A**) Strain correlation matrix based on alignment and average nucleotide identity (ANI). The distance was calculated using the Euclidean algorithm, and the linkage criterion utilized was complete linkage. The color bar below represents the percentage of identity between strains. (**B**) Evolutionary history inferred using the neigh-bor-joining method with the bootstrap consensus from 1000 replicates. The evolutionary distance was computed using the Jukes–Cantor method. Whole-genome alignments and the phylogenetic analyses utilized 25 selected pseudomonad genomes listed in Table S2. *Aeromonas salmonicida* J223 was used as an outgroup.

Twenty-five genomes of selected pseudomonads were aligned (Table S2). The ANI analysis was conducted on this alignment in CLCGWB platform to compare the pseudomonad genomes. This genome-based ANI analysis revealed that *P. libanensis* DMSP-1 and *P. lactis* SS101 share significantly higher percent identity (~95%) with *Pseudomonas* sp. J380 (Figure S6). Moreover, *Pseudomonas* sp. J380 demonstrated ~84–~87% of identity with other *P. fluorescens* strains analyzed. A correlation matrix showed that *Pseudomonas* sp. J380 clustered together with *P. libanensis* DMSP-1 and *P. lactis* SS101 (Figure 5A). The genome-based phylogeny analyses using CLCGWB (Figure 5B) and MEGA (Figure S7) also positioned *Pseudomonas* sp. J380 within a sub-cluster together with *P. libanensis* DMSP-1 and *P. lactis* SS101 with 100% bootstrap support.

### 3.8. Transcriptome Profile of Pseudomonas sp. J380 under Iron-Enriched and -Limited Conditions

#### 3.8.1. Global Profile of DEGs

**Figure 6:**
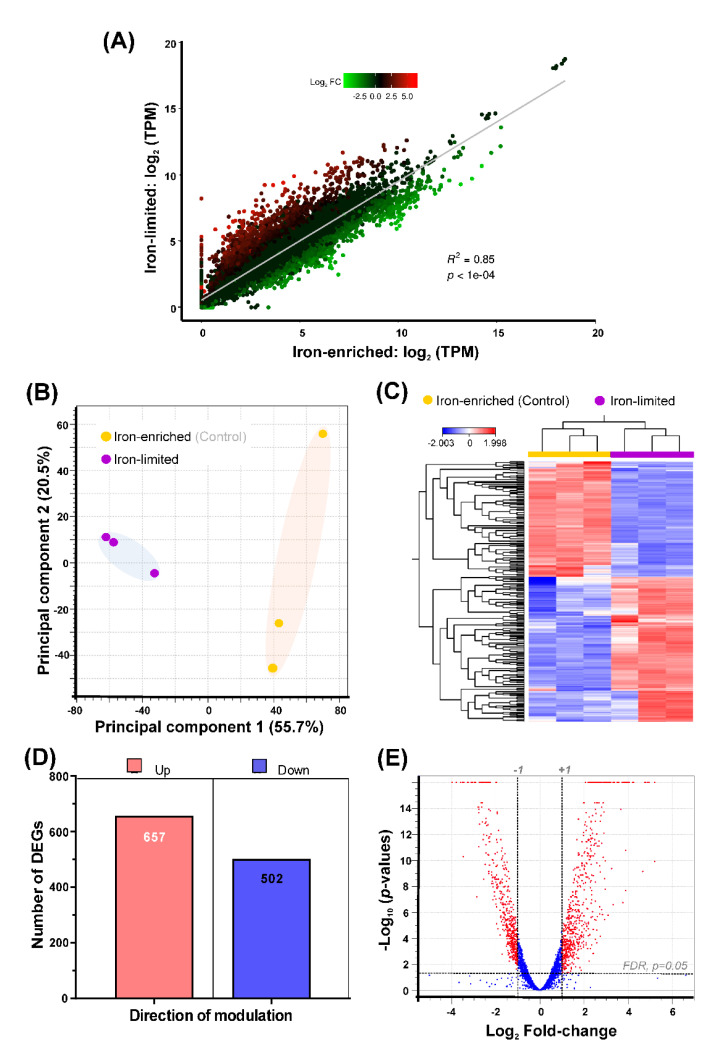
Global transcriptomic profiling of *Pseudomonas* sp. J380 by RNA-Seq. A total of six RNA libraries comprising three biological replicates for two different (iron-enriched versus iron-limited) conditions were included in the RNA-Seq experiment. (**A**) Scatter plot of RNA-seq expression under iron-enriched and iron-limited conditions. Each dot represents a gene; where red, green and black represent up-, down-regulated and non-differentially expressed genes. (**B**) Principal component analysis (PCA) of bacterial samples from iron-enriched and iron-limited conditions based on the expression of all datasets. (**C**) Heat map clustering of differentially-expressed genes (DEGs); color bars below the horizontal cluster indicate control (iron-enriched, yellow) and experimental (iron-limited, purple) samples. (**D**) The number of biologically significant DEGs with log_2_ FC ≥ |1| and FDR, *p* ≤ 0.05. (**E**) Volcano plot of DEGs; red dots, significant DEGs.

In order to study the physiological and environmental adaptation mechanisms of *Pseudomonas* sp. J380 under iron-stress, we determined the global gene expression profile under iron-enriched and iron-limited conditions using RNA-Seq. Information regarding the sequencing statistics and data quality is provided in Figure S2 and Table S3. A global expression correlation analysis showed a high degree of expression correlation (R^2^ = 0.8524; *p* < 0.0001) between iron-enriched vs. iron-limited samples (Figure 6A). PCA results and heat map revealed clear segregation of samples based on growth conditions (Figure 6B,C). PC1 and PC2 explained 76.2% of the total variation in expression data (Figure 6B). Tight distribution of samples under iron-limited conditions compared to control samples indicated that iron has a global impact on gene expression. A clear separation of samples based on growth conditions as illustrated in the heat map was in agreement with the PCA results (Figure 6C). The log_2_ fold-change (FC) ≥ |1| and false discovery rate (FDR) *p-*value of ≤0.05 were set as the cut-off criteria for significant differential expression. We found 1159 differentially-expressed genes (DEGs) under iron-limited conditions compared to the iron-enriched condition. These DEGs included 657 up-regulated and 502 down-regulated genes (Figure 6D). Gene identifier, description/annotation, fold-change, FDR (*p*-value), and associated GO terms for the DEGs of *Pseudomonas* sp. J380 under iron-limited conditions compared to the iron-enriched conditions are listed in Table S5.

#### 3.8.2. Analyses of Gene Ontology (GO) Terms and KEGG Pathways

**Figure 7:**
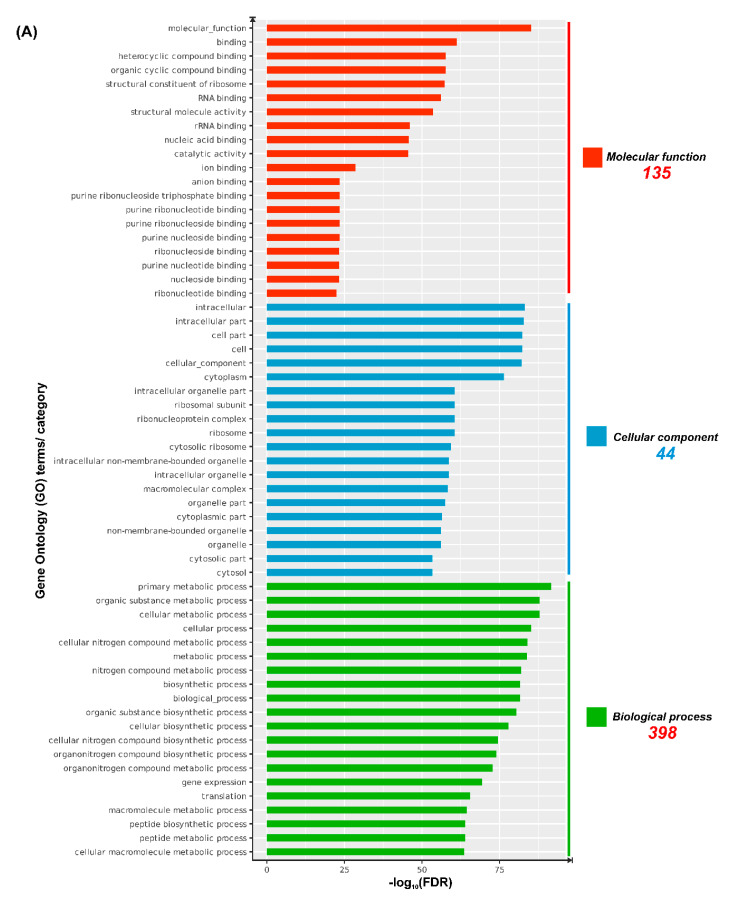

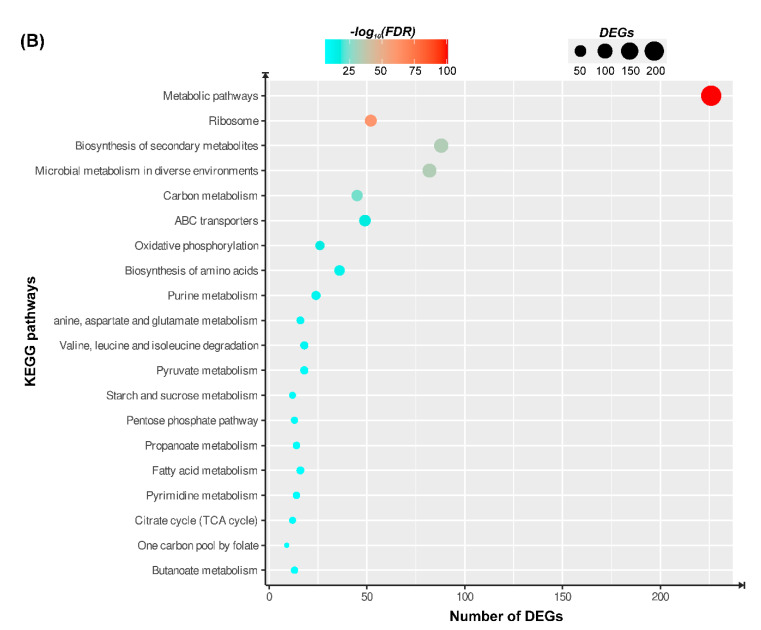
Enrichment analyses of *Pseudomonas* sp. J380 DEGs under iron-limitation. (**A**) Results from Gene Ontology (GO) enrichment analysis performed using ShinyGO illustrating twenty most significantly (*p* < 0.05) enriched GO terms under molecular function (MF), cellular component (CC), and biological process (BP) categories. (**B**) Results from KEGG (Kyoto Encyclopedia of Genes and Genomes) pathway enrichment analysis performed using KEGG database illustrating twenty most significantly (*p* < 0.05) enriched pathways. All the adjusted statistically significant values of GO terms or KEGG pathways were negative log_10_-transformed. For complete lists of enriched GO terms and KEGG pathways, refer to Tables S6 and S7).

In order to investigate which biological aspects are affected by these DEGs identified under iron-limited conditions, GO term and KEGG pathway analyses were performed. ShinyGO determined a total of 577 GO terms (MF, 135; CC, 44; and BP, 398) with an FDR *p*-value cut-off of 5% (Table S6). The twenty most significant GO terms from each GO category and KEGG pathways are graphically illustrated in Figure 7. It was evident that several GO terms associated with cellular metabolism, gene expression, and biosynthesis were enriched (Figure 7A). There were 62 enriched KEGG pathways (5% FDR), where a majority of them were related to metabolic processes (biosynthesis and degradation) (Figure 7B; Table S7).

Selected transcribed genes representing and playing roles in different cellular processes and their transcriptional changes under iron-limitation are tabulated in Table 3. A significant drop in the transcription of genes listed under enriched GO terms and KEGG pathways associated with basic cellular functions was evident from RNA-Seq results. Multiple ribosomal structural proteins (e.g., FXO12_13410, *rplM*; FXO12_22175, *rplF*; FXO12_24545, *rplS*; FXO12_14665, *rplY*; FXO12_26170, *rpmF*; FXO12_18260, *rpmH*; FXO12_11415, *rpsB*; FXO12_15685, *rpsF*; FXO12_22170, *rpsH*; FXO12_13405, *rpsI*; FXO12_14490, *rpsT*; and FXO12_21760, *rpsU*), elements or regulators involved in transcription (FXO12_23400, *greA*; FXO12_22365, *nrdR*; FXO12_22390, *nusB*; FXO12_19455, *rho*; FXO12_23460, *nusA*) and translation (FXO12_02255, *infA* and FXO12_00580, *infC*) were down-regulated under iron-limitation (Table 3 and Table S5). Components of respiratory chain (e.g., FXO12_26945, *ccoD*; FXO12_26935, *ccoP*; FXO12_24070, *cyoD*; FXO12_26950, *ccoN*; FXO12_08875, *ccmA*; FXO12_08720, *sdhC*) were also transcriptionally repressed (Table 3).

Increased transcription of genes encoding for elements of the two-component system (TCS), histidine utilization (Hut), and genes involved in defense against stress and oxidative damage was also observed (Table 3). The transcriptome analysis of *Pseudomonas* sp. J380 under iron-limited conditions showed dysregulation of 16 TCS genes. Among the genes encoding TCS components, seven sensor histidine kinases (HKs; e.g., FXO12_02805, FXO12_07360, FXO12_15195) and eight response regulators (RRs; e.g., FXO12_27685, FXO12_15180, FXO12_14850) were up-regulated, whereas 1 RR (FXO12_02395) was down-regulated. Histidine metabolism was among the enriched KEGG pathways, too (FDR = 1.38 × 10^–5^; Table S7), and the Hut pathway-associated genes were found to be up-regulated. Coincidently, a complete *hut* operon (*hutCDUH2H1G*) was identified in the *Pseudomonas* sp. J380 genome (Figure S8). Two copies of *hutH* (FXO12_16485, FXO12_16490) and a *hutU* (FXO12_16515) were found up-regulated under iron-limited conditions. In addition, out of six genes encoding mediators of stress and oxidative damage, five were up-regulated (i.e., FXO12_25080, *dps*; FXO12_02520; FXO12_06285; FXO12_17910, *katE*; FXO12_13240, *sod*), whereas FXO12_18785 (rubredoxin) was down-regulated (Table 3).

**Figure 8:**
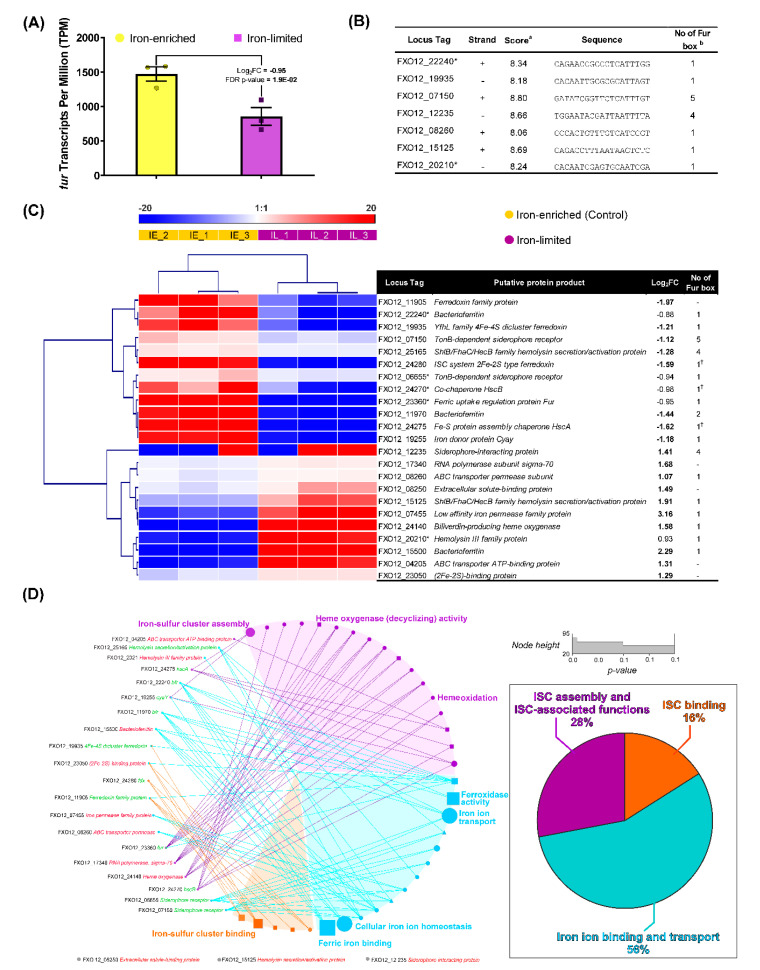
Putative elements associated with iron homeostasis in *Pseudomonas* sp. J380 identified by RNA-Seq under iron-limitation. (**A**) Number of *fur* (Ferric uptake regulation protein; FXO12_23360) transcripts under iron-enriched and iron-limited conditions. (**B**) Selected genes harboring predicted Fur box sequences. a, predictive score provided by regulon analyses using Virtual Footprint tool [50]. This inset table only shows Fur boxes with a score >8.0; b, when a gene contains more than one Fur box, the one with the highest score is shown. (**C**) Heat map illustration of genes with the putative function in iron homeostasis. †, part of the *isc* operon (Figure S12); -, no Fur boxes detected; FC of DEGs is in bold. *, refer to Table 3 footnote. (**D**) Gene Ontology (GO) term enrichment and pathway term network analysis of genes associated with iron homeostasis. GO enrichment analysis was performed with ClueGO [48]. Inset pie chart summarizes % genes per GO group. The circular pictogram is divided into left and right halves for genes and GO terms, respectively, in which the functionally grouped GO terms share the same color. The shape of the nodes indicates specific GO modules (i.e., ellipse, BP; triangle, MF; rectangle, CC). Names of the up- and down-regulated genes are indicated in red and green, respectively. As shown in the legend, node height reflects the term *p*-value corrected with Bonferroni step down method. Three genes that are not connected with any GO terms are shown below the circular pictogram. Refer to the File S1 for details.

Iron limitation altered several genes associated with iron homeostasis (Figure 8 and Table 4). Ferric iron uptake regulator (Fur), the master mediator of iron homeostasis, was slightly down-regulated under iron-limitation, and absent in the DEG list (FXO12_23360; Log₂FC = −0.954; FDR = 0.019; Figure 8A). Moreover, many of these DEGs harbored putative Fur box sequences in their promoter regions (Figure 8B,C). There was a Fur box in the promoter of *fur* itself (Figure 8C).

A pair of hemolysin secretion/activation proteins (FXO12_25165, FXO12_15125) with putative iron-liberating function demonstrated contrasting transcription profiles under iron-limitation. Putative proteins involved in siderophore-mediated iron piracy, including sigma-70 ECF factor (FXO12_17340), siderophore-interacting protein (FXO12_06740) were transcriptionally up-regulated. Although a TonB-dependent siderophore receptor (FXO12_07150) was less -expressed, a number of transport system involved in iron/iron-complex transportation (e.g., permease, FXO12_07455; Fe^3+^-transporter (FXO12_08250) and two ABC transporters, FXO12_04205, FXO12_08260) were over-transcribed. Genes encoding a biliverdin-producing heme oxygenase (*bpho*; FXO12_24140) and an iron-storing bacterioferritin (FXO12_15500) were also found to be highly expressed. In addition, the transcript abundance of several genes encoding proteins related to iron-sulfur cluster (ISC), including *hscA* (FXO12_24275) and *fdx* (FXO12_24280), two other putative *fdx*-like genes (FXO12_11905, FXO12_19935) and *cyaY* (FXO12_19255) decreased under iron-limited conditions (Figure 8C and Table 4). Interactive analysis of GO terms and genes involved in iron homeostasis identified three GO clusters including ‘ISC binding’, ‘ISC assembly and ISC-associated functions’, and ‘iron ion binding and transport’ (Figure 8D and File S1). Each GO cluster was composed of specific GO terms that interacted with one or multiple genes (Figure 8D).

## 4. Discussion

Based on previous studies, cunners have been considered as potential sea lice cleaner fish for the Atlantic salmon aquaculture industry, and the development of a breeding program towards establishing cunner hatcheries in Canada has recently been suggested [51]. Over the past few years, wild-caught cunners captured along the NL coastal waters by the field service team of the DOS, MUN, have often exhibited characteristic skin lesions and ulcers. Unfortunately, infectious diseases impacting cunner fish are not well characterized and documented [52]. In this study, we report the identification and complete characterization of a novel Gram-negative marine pathogen infecting wild cunners.

### 4.1. Characterization of Phenotypic, Physiological, and Biochemical Features

*Pseudomonas* sp. J380 was isolated from the head kidney, liver, and spleen of infected wild cunners, and we found that all the strains isolated showed identical characteristics (Table 1). This also indicated that *Pseudomonas* sp. J380 is capable of colonizing multiple internal organs of the cunners during a natural infection.

The halotolerant nature of *Pseudomonas* sp. J380 indicated that this bacterium is adapted to marine environments. *Pseudomonas* sp. J380 was capable of tolerating a wide temperature range (4–28 °C). This mesophilic property and inducible pyoverdine synthesis at higher temperatures (i.e., 28 °C) suggested that increasing ocean temperatures due to climate change might favor the infection events caused by *Pseudomonas* sp. J380.

The API20NE analysis suggested *P. fluorescens* (99.4% of confidence) as the significant taxa for the strain isolated in the current study. Being one of the diverse groups within the *Pseudomonas* genus, *P. fluorescens* species complex comprises more than 50 species, which have been found in various habitats including marine water [53]. In agreement with the phenotypic characterization, the majority of the substrate utilization and enzyme characteristics of *Pseudomonas* sp. J380 were similar to those reported from other *P. fluorescens* strains (e.g., CFS215 and 3a) [54,55]. Similar to these strains, *Pseudomonas* sp. J380 demonstrated detectable activities for catalase, urease, acid and alkaline phosphatases, and arginine dihydrolase, and capacity for exploiting various C-sources such as glucose, mannose, and maltose [54,55].

*Pseudomonas* spp. have been reported to carry multiple intrinsic and acquired antimicrobial resistance genes [56]. The genome of *Pseudomonas* sp. J380 was found to harbor putative antibiotic resistance genes associated with the observed phenotypic antibiogram (Table 1). Two copies of *dhfr* (*dfrA*; FXO12_02730, FXO12_19880) and a single copy of *folP* (*sul* or *dhps*; FXO12_23425) that encode for dihydrofolate reductase and dihydropteroate synthase, respectively, were identified and could be responsible for SXT-resistance [57,58]. An *ampC* gene (FXO12_03565) encoding a class C beta-lactamase, mediating AMP- and CTX-resistance, as well as a putative *ppqFABCDE* operon responsible for pyrroloquinoline quinone biosynthesis (Figure S9) and implicated with CHL-resistance, were also present in *Pseudomonas* sp. J380 genome [59,60].

Siderophores are iron-scavenging molecules produced by microbes and the species that produce siderophores survive better in iron-limited environments [61]. In addition, siderophores act as virulence factors in many bacterial pathogens including pseudomonads [62]. We showed that *Pseudomonas* sp. J380 synthesized fluorescent siderophore, which is known as pyoverdine. These high-affinity fluorescent siderophores are mainly produced by *P. fluorescens* species complex [63]. In addition to their iron uptake role, pyoverdines have been implicated with the colonization of host tissues and the formation of biofilms [64]. Confocal microscopic observation of *Pseudomonas* sp. J380 further confirmed the pronounced production of pyoverdines under iron-limited conditions compared to iron-enriched conditions (Figure 1). Based on this pyoverdine-secreting property of *Pseudomonas* sp. J380, we hypothesized that it could be a novel member of the *P. fluorescens* species complex.

### 4.2. Characterization of Infectivity and Host-Specificity

We used the *Pseudomonas* sp. J380 isolated from infected wild cunners to verify the Koch’s postulates. At the end of the infection assay, the survival of cunners in the low and medium dose groups was similar (~88%), and it decreased up to 79% in the high dose group. The clinical signs observed during the disease progression and the biochemical characteristics of the re-isolated bacterium were similar to those of the original strain from wild cunner fish. Next, we expanded our infection assays with lumpfish and Atlantic salmon to determine if *Pseudomonas* sp. J380 could infect different hosts. Although the mortality started at almost the same time (5–6 dpi), percent survival varied between species. Based on the percent survival, it was interesting to note that *Pseudomonas* sp. J380 presents a more acute virulence in lumpfish when compared with cunners. This observation raised a negative concern over the cohabiting cunner-lumpfish dual-cleaner fish model in sea cages since cunners could horizontally transmit *Pseudomonas* sp. J380 to lumpfish, which could end up in mass mortality of both cleaner fish species. Our results revealed that *Pseudomonas* sp. J380 infected both cunners and lumpfish, but not Atlantic salmon.

As a part of determining Koch’s postulates as well as to study the infection kinetics, *Pseudomonas* sp. J380 was re-isolated from multiple internal organs of infected fish. The degree of bacterial colonization varied in terms of host species, organs, and time (dpi). Immune tissues of cunners (liver, spleen, and head kidney) were heavily colonized at initial and later (7 and 35 dpi) phases. Conversely, low relative bacterial load was detected in lumpfish (except for 14 dpi) and Atlantic salmon. Our data suggested that lumpfish is the most susceptible species to *Pseudomonas* sp. J380 among the hosts examined, regardless of the low bacterial colonization. This was in agreement with our post-mortem findings (Figure S4C,D,F). In contrast to lumpfish, cunners seem better at confronting the pathogen as revealed by relatively low mortality despite the heavy tissue colonization, and severe damage in multiple organs (Figure S4B,E,G). The low virulence of *Pseudomonas* sp. J380 combined with its persistence suggests that *Pseudomonas* sp. J380 and cunners have been interacting for a long time, and *Pseudomonas* J380 might be an opportunistic pathogen in these animals. However, under stress conditions, for instance, after the hypometabolic dormancy in spring, some cunners may become immune suppressed and susceptible to *Pseudomonas* sp. J380 resulting in chronic infection. Additionally, we cannot rule out that the wild cunners utilized in our experiments might have developed immunity against *Pseudomonas* sp. J380, thus less-susceptible.

### 4.3. Characterization of Genomic Features, Comparative, and Phylogenomics

The *Pseudomonas* sp. J380 genome was composed of a single circular chromosome of 6.26 Mbp, encoding 5568 putative proteins. There were 808 (14.5%) hypothetical genes with no assigned function. Among the ncRNAs found in the *Pseudomonas* sp. J380 genome, three were encoded by *rnpB*, *ssrS* and *ffs*, and their regulatory roles remain unknown. The size and GC% content of *Pseudomonas* sp. J380 were comparable to those of different pseudomonads (Table S2). By homology search, we found ~70 virulence factors encoded by *Pseudomonas* sp. J380 genome (Table S4), however, their roles in physiology and pathogenesis require further investigation.

We utilized the genetic information to phylogenetically classify *Pseudomonas* sp. J380. First, we attempted to confirm the identity of the isolated strain based on the sequences of eight canonical genes from different pseudomonads using MLSA-based phylogeny. Our results suggested that *Pseudomonas* sp. J380 belongs to the *P. fluorescens* phylogroup. Next, we extended our analyses with 25 pseudomonad genomes using comparative genomic and phylogenetic approaches to validate our results. The closest relatives of *Pseudomonas* sp. J380 were *P. libanensis* DMSP-1 and *P. lactis* SS101, both of which are members of *P. fluorescens* phylogroup. In addition, the NCBI database indicates that *Pseudomonas* sp. J380 belongs to unidentified *Pseudomonas* species. These outcomes strongly suggest that *Pseudomonas* sp. J380 is a novel strain belonging to *P. fluorescens* species complex.

### 4.4. Profile of Iron-Regulated Transcriptome

Iron acquisition strategies play vital roles in the growth, pathophysiology, and virulence of pathogenic bacteria [22]. In order to successfully colonize the host and to establish the infection, pathogenic bacteria have to overcome a transient period of iron limitation by scavenging iron from different host sources [65]. As a part of their nutritional immunity, hosts employ multiple iron sequestering mechanisms to tightly regulate the availability of iron for pathogens. However, bacterial pathogens have evolved several transcriptionally regulated iron acquisition strategies to circumvent these withholding strategies of hosts [22,23]. Identifying the elements of these mechanisms will certainly provide clues about how *Pseudomonas* sp. J380 colonizes host tissues and further sheds light on its pathogenicity. We examined the global transcriptomic response of *Pseudomonas* sp. J380 under iron-enriched and iron-limited conditions using RNA-Seq to identify the potential DEGs, and to explore the associated biological processes.

Our RNA-Seq analyses revealed that ~1/5 (20.8%; 1159/5568) of the genes were dysregulated by iron-limitation, which included six pseudogenes. There were more over-expressed genes compared to less-expressed (Figure 6D,E). To explore the significantly impacted biological pathways by the bioavailability of iron, we conducted enrichment analyses using GO terms and KEGG pathways.

#### 4.4.1. Fundamental Cellular Functions

Two inter-related GO terms frequently represented by the DEGs identified in this study are ribosome (GO:0005840) and translation (GO:0006412) (Figure 7A). An array of genes encoding large (50S, *rpl*; *n* = 23) and small (30S, *rps*; *n* = 20) ribosomal proteins were all found to be less-expressed under iron-limitation (Table S5). As documented in previous studies [66,67,68,69], the overall down-regulation of ribosomal protein-coding genes, and other elements involved in transcription, such as regulator (NrdR) and terminator (Rho), and translation, including elongation factors (e.g., EF-G and EF-P) and translation initiation factors (IF-1−3), combined with the energy deficiency, could diminish protein synthesis and retard growth of *Pseudomonas* sp. J380 under iron-limitation. The translation is an energy-demanding process, and therefore protein biosynthesis is strongly correlated with respiration, energy production, and metabolism [66].

Oxidative phosphorylation, the final step of respiratory metabolism in aerobic organisms, typically utilizes iron cofactor-dependent proteins and was present in some enriched GO terms (GO:0019646). Genes encoding members of different cytochrome subunits were found to be differentially expressed under iron-limitation. Two cytochrome-c oxidase-related genes (FXO12_17965, FXO12_17960), that are arranged in a putative cytochrome c oxidase cluster along with *ctaD* and *coxB*, were up-regulated (Log_2_FC = 1.16; Table S5). However, the majority of them were down-regulated (e.g., *ccoN*, *cyoD*, *ccoP*) suggesting that iron limitation hindered energy metabolism in *Pseudomonas* sp. J380 similar to other species [68,70] (Table 3 and Table S5). Respiratory complex II is composed of multiple succinate dehydrogenase (SDH) iron-sulfur proteins and a vital enzyme participating in both Kreb’s cycle and electron transport chain (ETC). *Pseudomonas* sp. J380 possesses four genes in a putative *sdhCDAB* cluster (Figure S10A; *sdhB*, FXO12_08710, *sdhD*, *sdhC*), in which the last two genes were down-regulated by iron-limitation. The *atp* operon of *Pseudomonas* sp. J380 is composed of eight genes (*atpBEFHAGDC*) encoding for subunits of ATP synthase (Figure S10B), and all of them were down-regulated (log_2_FC −1.11 to −2.23) under iron-limitation. In agreement with previous results, the decreased transcription of genes involved in Kreb’s cycle and ETC implied that respiratory metabolism was inhibited by iron-limitation in *Pseudomonas* sp. J380. However, we also noticed an increased expression of glycolytic enzymes (e.g., *glk*, *pgi*; Table S5) involved in alternative ATP production to potentially compensate for the decreased energy generation from Kreb’s cycle and ETC [71].

#### 4.4.2. Two-Component Systems (TCS)

TCS are dominant gene control switches in bacteria and play essential roles in signal transduction, physiology, cell-cell communication, adaptation to changing environments, host interaction, and pathogenesis [72]. Classical TCS consist of a sensor HK that phosphorylates the cognate RR upon receiving a signal. RRs are generally DNA-binding transcriptional modulators and could activate or repress their target genes. By controlling the expression of ferrienterobactin receptor that involves in iron uptake, TCS was found to take part in iron homeostasis of *P. aeruginosa* [73]. Transcriptomic analyses showed the increased expression of 4 out of 7 HKs by log_2_FC > 2 and 8 RRs by log_2_FC > 1 (Table 3). Conversely, an OmpR domain-containing RR was down-regulated (FXO12_02395). Up-regulated TCS encoding genes under iron-limitation have also been reported in other pseudomonads [70,74]. The increased expression of multiple TCS components in the current study indicated that *Pseudomonas* sp. J380 TCSs may govern an array of downstream genes in response to low iron levels. Nevertheless, pairing HKs to their cognate RRs, and their relation to iron-mediated transcriptional regulation (e.g., Fur-mediation) require further functional investigation in *Pseudomonas* sp. J380.

Although nearly 70 putative virulence factors encoding genes were identified in *Pseudomonas* sp. J380 genome, majority of them were not differentially expressed (except LPS biosynthesis protein, FXO12_08045; Flp family type IVb pilin, FXO12_15135; *cpaB* and *cpaF*) under iron-limitation (Table S4). Over-production of alginate could promote survival and persistence of *Pseudomonas* spp. by making them a mucoid phenotype and alginate is considered to be a virulence factor [75]. The components of the alginate biosynthetic pathway in *P. aeruginosa* were encoded by *alg* operon [76]. We identified the 12-gene *alg* operon in *Pseudomonas* sp. J380 genome by homology search (Figure S11). While *alg* operon was not differentially expressed, a few regulators of this operon, including *algR*, (FXO12_19295), *algP* (FXO12_19335), *algB* (FXO12_17830), *kinB* (FXO12_17835) were determined to be up-regulated by iron-limitation.

#### 4.4.3. Hut Pathway

The Hut pathway is associated with histidine catabolism and utilizes several enzymes. The genes encoding these histidine metabolizing enzymes are generally structured in a conserved operon [77]. *Pseudomonas* sp. J380 genome possessed 6 Hut pathway genes (Figure S8; two copies of *hutH*, each one copy of *hutU*, *hutG*, *hutD*, *hutC*), where both copies of *hutH* and *hutU* were induced under iron-limited conditions. Histidine metabolism and Hut pathway have been implicated in bacterial virulence and pathogenesis [78,79]. Production of extracellular proteases in *V. alginolyticus* was found to utilize the Hut pathway [78]. Moreover, HutC has been shown to directly regulate the expression of virulence-related genes [79]. Although *hutC* repressor had no significant transcriptional changes in our study, there might be potential changes in virulence of *Pseudomonas* sp. J380 as previous studies suggested the existence of alternative regulators (e.g., TCS) that could serve as a transcriptional activator of *hut* genes [80].

#### 4.4.4. Stress and Oxidative Damage-Related Genes

Transcriptional modification occurred in genes encoding proteins that are involved in oxidative stress protection (Table 3). Synergistic induction of two classic redox biomarkers *katE* (catalase) and *sodA* (MnSOD) was noticed. These oxidative stress-responsive enzymes counteract against harmful reactive oxygen species (ROS). Iron-limitation induced the expression of *dps,* a gene that encodes for a stress-mediatory protein with dual functions, including protecting DNA from oxidative damage and ferroxidase activity [81]. Dps oxidizes Fe^2+^ at its ferroxidase center and stores the resulting Fe^3+^ within the iron core [82]. Hemerythrin (Hr) is a nonheme Fe-containing O_2_ carrier-protein found in marine organisms [83]. Hr-like domain-containing proteins in prokaryotes are thought to perform secondary functions including O_2_ sensing [84] and protecting from oxidative damage [85]. Meanwhile, elevated expression of an Hr domain-containing protein (FXO12_06285) and a putative iron-containing redox protein (FXO12_02520; member of haem oxygenase (HO) superfamily) was detected under iron-limitation. Modulated transcription of redox genes has also been observed in other bacteria under iron-limited conditions [70,86]. Under low intracellular iron levels, the probability of accumulating harmful levels of ROS is low. However, the increased expression of oxidative stress responders in *Pseudomonas* sp. J380 suggests that their transcriptional regulation might be under the governance of Fur [70]. Rubredoxin (Rd) is an iron-containing multifunctional protein, and it forms an alternative cytoplasmic oxidative stress protection system along with rubrerythrin [87,88]. Among two putative *rd* orthologs found in *Pseudomonas* sp. J380 genome, one was significantly less transcribed under low iron levels in the current study (Table 3).

#### 4.4.5. Iron-Homeostasis

Our DEG profiling revealed the transcriptional modulation of various proteins involved in iron-homeostasis (e.g., acquisition, transportation, and storage) and several iron-containing (e.g., Fe-S) proteins. Proteomic profiling of *Yersinia ruckeri* under normal and iron-limited conditions revealed differential expression of an array of proteins involved in Fe^3+^ capture and transport, and cellular metabolism [69].

The majority of iron acquisition strategies in bacterial pathogens, including pseudomonads, are completely or at least partially regulated by members of the Fur superfamily [89,90]. Fur is a homodimeric metalloregulator that binds to Fur box within the promoter region of target genes when liganded by Fe^2+^, and inhibits the transcription under iron-enriched conditions [22]. Fur repressor dissociates from DNA when Fe^2+^ level is low allowing transcription of target genes to occur. As a global regulator of iron uptake, it directly and indirectly controls the expression of associated regulatory RNAs, sigma factors, and various transcriptional regulators [90]. Fur was found to be down-regulated in our study (Figure 8A) suggesting that many potential Fur-regulated target genes could have actively transcribed under iron-limited conditions. Regulon prediction analysis showed that majority of the DEGs involved in iron homeostasis harbor putative Fur box sequences (Figure 8B,C). The auto-regulatory feature of Fur has been previously reported in a few species [91]. It was interesting to note the presence of Fur box at the promoter of *fur* itself, suggesting that Fur might regulate its own expression in *Pseudomonas* sp. J380; however, this requires further investigation.

The fundamental mechanisms for iron uptake by pathogenic bacteria include (1) sequestering heme-Fe from host hemoproteins utilizing receptors and secreted proteins, (2) capturing Fe^3+^ from host iron-storages (e.g., transferrin and lactoferrin) using binding proteins and siderophores, and (3) permeabilizing free iron with the aid of ferric reductases and ferrous permeases [22]. Heme is a preferred host iron source for bacteria that secrete hemolysins to enhance the heme availability. *Pseudomonas* sp. J380 genome possesses five putative hemolysin-related elements (Table S8), and two of them (FXO12_25165, FXO12_15125; hemolysin secretion/activation protein) were present in the DEG list.

A number of genes involved in the biosynthesis of siderophores including pyoverdines have been reported from different pseudomonads [92]. In *P. aeruginosa*, *pvcABCD* operon is involved in pyoverdine synthesis, while *pvdD*, *pvdS* and *pvdR* are involved in other regulatory roles. Based on current annotation, a few genes associated with pyoverdine siderophores were identified in *Pseudomonas* sp. J380 genome (e.g., FXO12_27210, siderophore synthetase; FXO12_12235, siderophore-interacting protein; FXO12_06740, PvdJ/PvdD/PvdP-like protein; Table S8). Moreover, the *Pseudomonas* sp. J380 genome has 18 TonB-dependent siderophore receptor genes and three of them were DEGs (Table S8). These receptors facilitate uptake of iron in the form of siderophore- or other protein-complexes. The sigma ECF factors in different species have been implicated in the regulation of heat-shock response, iron-transport, metal ion efflux system, and alginate secretion [93]. A sigma-70 ECF factor (FXO12_17340) was up-regulated in *Pseudomonas* sp. J380 under iron-limitation; however, its association with iron-homeostasis remains to be characterized. Unexpectedly, only a few genes involved in putative siderophore biogenesis and uptake were identified as DEGs, including FXO12_12235 and a TonB-dependent siderophore receptor (FXO12_07150). This implied that alternate pathways for these molecular events might be existing in *Pseudomonas* sp. J380. Soluble Fe^2+^ can freely enter through the outer membrane of Gram-negative bacteria, and from the periplasm, Fe^2+^ transport occurs through metal uptake transporters [94]. Transcript of Fet4-like iron permease (FXO12_07455) was strongly up-regulated (log_2_FC = 3.16). Two up-regulated ABC transporter family members and EC solute binding ferric transporter (FXO12_08250) might take part in iron transportation. In accordance with earlier studies [70,74,95], enhanced transcription of various iron transporter systems in *Pseudomonas* sp. J380 under iron-starvation suggests that it strives to effectively modulate the intracellular iron levels.

Once the heme reaches the bacterial cytosol, heme oxygenases (HOs) release Fe^2+^ by catabolizing the heme to produce biliverdin and CO [96]. Transcript of a BPHO homolog (FXO12_24140) was induced by iron limitation. Similarly, HO orthologs have been shown to be abundantly expressed under iron limitation in different pseudomonads [68,74,97], further suggesting a pivotal iron acquisition role for HOs in bacteria during iron starvation.

There are two types of ferritin-like iron storage molecules found in bacteria: bacterial ferritins (Ftn) and bacterioferritins (Bfr) [98]. We identified three putative *bfr* genes in the genome of *Pseudomonas* sp. J380. While one was induced by iron limitation (FXO12_15500), the other two that shared ~45% amino acid identity were down-regulated. Protein sequence-wise, the upregulated *bfr* was quite different from down-regulated *bfr*s. Similar results were reported in *P. aeruginosa* and *P. fluorescens* Pf-5 [70,95], suggesting a reduced need for iron storage when iron availability is low. Bacterioferritin-associated ferredoxin (Bfd) is a bacteria-specific physiological cognate partner of Bfr whose function is to mobilize and release iron from Bfr. However, transcription of a *bfd* ortholog (FXO12_11965) in *Pseudomonas* sp. J380 remained unaffected under iron limitation, presumably to sustain the iron storage within Bfr.

Fe-S cofactors are utilized in several molecular functions such as electron transfer and activation of substrates to sense ROS. A family of metalloproteases containing Fe-S cluster takes part in vital cellular functions including ribosome function, amino acid and C metabolism, and transcription [99]. To assemble these clusters, bacteria employ Fe-S cluster biogenesis systems, such as the iron-sulfur cluster (ISC) pathway. The enzymatic Fe-S cluster machinery is encoded by *isc* operon, which is composed of eight genes in the classic *Escherichia coli* model [100]. We identified a putative *iscRSUA-hscBA-fdx-iscX* cluster in *Pseudomonas* sp. J380, which was structurally identical to that of *E. coli isc* operon (Figure S12). Although the genes in *isc* cluster were found to be down-regulated (except *iscX*), only *hscA* and *fdx* were identified as significantly DEGs in our study. While HscA (and its co-chaperon HscB; FXO12_24270) is involved in ISC assembly or transfer [101], Fdx acts as an electron donor in the ISC biogenesis pathway [99]. Two other putative *fdx*-like genes (FXO12_11905 and FXO12_19935) and an iron-trafficking CyaY protein that supplies iron for ISC biogenesis were also less-expressed under iron-limitation. In contrast, a 2Fe-2S ISC binding domain-containing protein (FXO12_23050) was up-regulated. Not surprisingly, the promoter region of *iscR* was found to harbor a putative Fur binding site suggesting a potential iron-dependent, Fur-mediated transcriptional regulation of *isc* operon in *Pseudomonas* sp. J380. These findings suggest that iron limitation depressed the *isc* operon resulting in an overall reduction in the biogenesis of Fe-S proteins, which could lead to compromised cellular processes that depend on Fe-S proteins including genome maintenance, protein translation, and energy conversion.

To visualize the interaction between GO terms and genes associated with iron homeostasis, we used ClueGO and determined three GO clusters including ‘ISC binding’, ‘ISC assembly and ISC-associated functions’, and ‘iron ion binding and transport’ (Figure 8D and File S1). The information on bacterial gene annotation was a significant limiting factor. The identification of more iron homeostasis-related genes will depend on the curated annotation of homologs, and advances in omics resources will help to resolve this.

## 5. Conclusions

In this study, the causative agent of a natural infection in wild cunners in Atlantic Canada was isolated and identified as a novel member of *Pseudomonas* species. *Pseudomonas* sp. J380 appears to be an opportunistic pathogenic bacterium in cunners and may emerge as an opportunistic pathogen when cunners are immune-suppressed resulting in chronic infection. However, lumpfish were more susceptible than cunners and acutely infected by *Pseudomonas* sp. J380. Under iron limitation, *Pseudomonas* sp. J380 produced pyoverdine-type siderophores. A large-scale transcriptomic alteration occurred under iron-limited conditions in various cellular processes, such as metabolism, TCS, Hut pathway, redox balance and, particularly, in iron-homeostasis. This study provides the basis for the biology of *Pseudomonas* sp. J380 and further studies are required to diagnose, manage and treat this disease.

## Figures and Tables

**Table 1:** Phenotypic characteristics of *Pseudomonas* sp. J380 strain isolated from wild cunner fish (*Tautogolabrus adspersus*).

Phenotypic Characteristics	Strain J380
Gram stain	−
Capsule	−
Cell shape	Rod
Motility	+
Oxidase	+
O-129	Resistant
Type-I fimbria	−
Hemolysis activity	+ (β-hemolysis)
Fluorescent siderophores	+
Catalase	+
***Growth characteristics***	
Growth at 4 °C	+
Growth at 15 °C	++
Growth at 28 °C	+++
Growth at 37 °C	−
Growth in LB 0% NaCl	++
Growth in LB 1% NaCl	++
Growth in LB 2% NaCl	++
***API20NE***	
Profile reference and prediction	0347554; 99.4% confidence, *P. fluorescens*
Reduction of Nitrates	−
Indole production	−
Fermentation of D-glucose	−
Arginine dihydrolase	+
Urease	+
Esculin Hydrolysis (β-glucosidase)	−
Gelatin Hydrolysis (protease)	−
β-galactosidase	−
Assimilation of:	
D-glucose	+
L-arabinose	+
D-mannose	+
D-mannitol	+
N-acetyl-glucosamine	+
D-Maltose	−
Potassium gluconate	+
Capric acid	+
Adipic acid	−
Malate	+
Trisodium citrate	−
Phenylacetic acid	−
***API ZYM***	
Alkaline phosphatase	+
Esterase (C-4)	+
Esterase Lipase	+
Lipase (C-14)	+
Leucine arylamidase aminopeptidase	+
Valine aminopeptidase arylamidase	+
Cystine aminopeptidase arylamidase	−
Trypsin	−
α-Chymotrypsin	−
Acid phosphatase	+
Naphthol-AS-BI-phosphohydrolase	+
α-galactosidase	−
β -galactosidase	−
Β-glucuronidase	−
α-glucosidase	−
β-glucosidase	−
N-acetyl-β-glucosaminidase	−
α-mannosidase	−
α-fucosidase	−
***Antibiotic resistance (Antibiogram)***	
Oxytetracycline (OTC)	– (19 mm) (Susceptible)
Tetracycline (TET)	– (21 mm) (Susceptible)
Oxolinic acid (OXA)	– (15 mm) (Susceptible)
Trimethoprim-sulfamethoxazole (SXT)	+ (Resistant)
Cefotaxime (CTX)	+ (Resistant)
Ampicillin (AMP)	+ (Resistant)
Chloramphenicol (CHL)	+ (Resistant)

For growth characteristics: +, scant; ++, moderate; +++, profuse.

**Table 2:** Summary of *Pseudomonas* sp. J380 genome.

Characteristics	In *Pseudomonas* sp. J380
Annotation Pipeline	NCBI, Prokaryotic Genome Annotation Pipeline (PGAP)
Annotation Method	Best-placed reference protein set; GeneMarkS-2+; v4.12
Genes (total)	5734
CDSs (total)	5642
Genes (coding)	5568
CDSs (with protein)	5568
Genes (RNA)	92
rRNAs	7, 6, 6 (5S, 16S, 23S)
complete rRNAs	7, 6, 6 (5S, 16S, 23S)
tRNAs	69
ncRNAs	4
Pseudo Genes (total)	74

**Table 3:** Selected differentially-transcribed genes playing roles in different cellular processes (except those functioning primarily in iron homeostasis).

Locus Tag ^a^	Region ^b^	Gene Symbol	Putative Protein Product ^c^	Log_2_ FC ^d^	FDR *p*-Value ^d^
***Ribosomal Proteins***					
FXO12_26170	5727130..5727312	*rpmF*	50S ribosomal protein L32 RpmF	**−3.48**	0
FXO12_13410	complement (2970861..2971289)	*rplM*	50S ribosomal protein L13 RplM	**−3.04**	0
FXO12_18260	4056006..4056140	*rpmH*	50S ribosomal protein L34 RpmH	**−2.79**	7.9 × 10^−15^
FXO12_21760	Complement (4790073..4790288)	*rpsU*	30S ribosomal protein S21 RpsU	**−3.24**	0
FXO12_14490	3182295..3182573	*rpsT*	30S ribosomal protein S20 RpsT	**−3.79**	0
***Protein Synthesis***					
FXO12_23400	5120014..5120490	*greA*	Transcription elongation factor GreA	**−1.09**	2.2 × 10^−3^
FXO12_22365	49025..4903169	*nrdR*	Transcriptional repressor NrdR	**−1.26**	3.3 × 10^−6^
FXO12_22390	4906674..4907174	*nusB*	Transcription antitermination protein NusB	**−1.39**	2.2 × 10^−5^
FXO12_19455	4322309..4323568	*rho*	Transcription termination factor Rho	**−1.41**	2.5 × 10^−4^
FXO12_23460	5128462..5129943	*nusA*	Transcription termination/antitermination protein NusA	**−1.82**	8.9 × 10^−8^
FXO12_02255	complement (448479..448697)	*infA*	Translation initiation factor IF-1	**−3.42**	0
FXO12_00580	95333..95866	*infC*	Translation initiation factor IF-3	**−1.47**	1.1 × 10^−4^
***Cellular Respiration***					
FXO12_26945	complement (5889113..5889721)	*ccoO*	Cytochrome-c oxidase, cbb3-type subunit II	**−1.11**	8.7 × 10^−3^
FXO12_26935	complement (5887965..5888912)	*ccoP*	Cytochrome-c oxidase, cbb3-type subunit III	**−1.25**	2.5 × 10^−3^
FXO12_24070	5267129..5267464		Cytochrome-o ubiquinol oxidase subunit IV	**−1.45**	9.1 × 10^−4^
FXO12_26950	complement (5889732..5891156)	*ccoN*	Cytochrome-c oxidase	**−1.79**	5.9 × 10^−5^
FXO12_08875	complement (2005507..2006142)	*ccmA*	Cytochrome-c biogenesis heme-transporting ATPase CcmA	**−2.11**	6.2 × 10^−9^
FXO12_08720	complement (1974082..1974456)	*sdhC*	Succinate dehydrogenase cytochrome b556 subunit	**−1.38**	3.4 × 10^−6^
***Two-Component Systems (TCS)***					
FXO12_02805	584990..586702		Histidine kinase	**3.13**	0
FXO12_07360	complement (1672777..1674333)		Histidine kinase	**2.53**	1.2 × 10^−14^
FXO12_15195	3323722..3326472		Histidine kinase (PAS domain-containing protein (serine HK))	**2.32**	0
FXO12_04765	complement (1011214..1012884)		Histidine kinase	**2.02**	5.3 × 10^−10^
FXO12_17835	3965314..3967101		Histidine kinase (alginate biosynthesis sensor protein KinB)^$^	**1.20**	2.8 × 10^−4^
FXO12_05065	complement (1084240..1085568)		Histidine kinase (HAMP domain-containing protein (HK))	**1.14**	3.9 × 10^−4^
FXO12_12155	2685187..2686566		Histidine kinase	**1.03**	1.4 × 10^−4^
FXO12_27685	complement (6046607..6047266)		Response regulator transcription factor	**2.50**	3.3 × 10^−11^
FXO12_15180	complement (3321856..3322641)		Response regulator transcription factor	**1.79**	3.1 × 10^−7^
FXO12_19295	4289028..4289774		Response regulator transcription factor	**1.69**	1.6 × 10^−5^
FXO12_17830	3963957..3965303		Response regulator	**1.40**	1.6 × 10^−4^
FXO12_14850	3247797..3248471		Heavy metal response regulator transcription factor	**1.25**	1.8 × 10^−3^
FXO12_02395	474143..474871		Response regulator transcription factor	**−1.04**	3.5 × 10^−3^
FXO12_19295	4289028..4289774	*algR*	DNA-binding response regulator (alginate biosynthesis regulatory protein AlgR)^$^	**1.69**	1.6 × 10^−5^
FXO12_19335	4296340..4297539	*algP*	Transcriptional regulator (alginate regulatory protein AlgP)^$^	**1.01**	4.4 × 10^−4^
FXO12_17830	3963957..3965303	*algB*	Response regulator (two-component response regulator AlgB)^$^	**1.40**	1.6 × 10^−4^
***Histidine Catabolism (Hut Pathway) ^e^***					
FXO12_16485	complement (3641498..3643036)	*hutH*	Histidine ammonia-lyase HutH_1	**1.13**	3.21 × 10^−3^
FXO12_16490	complement (3643063..3644571)	*hutH*	Histidine ammonia-lyase HutH_2	**1.37**	8.83 × 10^−5^
FXO12_16515	complement (3649120..3650790)	*hutU*	Urocanate hydratase HutU	**2.11**	2.82 × 10^−5^
FXO12_16470 *	complement (3637890..3638690)	*hutG*	N-formylglutamate deformylase HutG	0.64	3.04 × 10^−2^
FXO12_16520 *	complement (3651180..3651740)	*hutD*	HutD family protein	0.24	5.32 × 10^−1^
FXO12_16525 *	complement (3651737..3652486)	*hutC*	Histidine utilization repressor HutC	0.02	9.60 × 10^−1^
***Defense Against Stress and Oxidative Damage***					
FXO12_13240	complement (2935573..2936184)	*sodA*	Superoxide dismutase	**2.43**	1.5 × 10^−3^
FXO12_17910	complement (3981967..3984108)	*katE*	Catalase	**1.95**	1.4 × 10^−9^
FXO12_25080	5486878..5487351	*dps*	DNA-binding protein from starved cells (Dps)	**2.19**	5.5 × 10^−7^
FXO12_02520	497862..499241		Iron-containing redox enzyme family protein	**2.19**	0
FXO12_06285	1385806..1386282		Hemerythrin domain-containing protein	**1.63**	1.3 × 10^−9^
FXO12_18785	4178968..4179135	*rd*	Rubredoxin	**−1.14**	7.2 × 10^−5^

^a^, gene-specific identifier in NCBI genome database; ^*^ not present in the DEG list (as it does not satisfy the fold-change (log_2_FC > 1) and/or significance (FDR *p*-value < 0.05) threshold criteria. However, due to its importance in an associated functional category under which it has been listed, it is included here; ^b^, spanning of the coding sequence; ^c^, obtained from UniProt, NCBI, or *Pseudomonas* Genome DB (PGDB; https://pseudomonas.com/); ^$^, homology search was performed at PGDB using DIAMOND Blast against other pseudomonads; ^d^, data from RNA-Seq. FC of DEGs is in bold. For complete information of these transcripts, refer to Table S5; ^e^, refer to Figure S8 for gene structure of *hut* operon.

**Table 4:** Selected differentially-transcribed genes playing roles related to iron homeostasis.

Locus Tag ^a^	Region ^b^	Gene Symbol	Putative Protein Product ^c^	Log_2_FC ^d^	FDR *p*-Value ^d^	Conserved Domain ^e^	Putative Function ^f^
FXO12_23360 *	5108033..5108437	*fur*	Ferric uptake regulation protein Fur	−0.95	1.94 × 10^−2^	Fur	Master regulator of Fe^3+^ uptake
FXO12_25165	5500602..5502314		ShlB/FhaC/HecB family hemolysin secretion/activation protein	**1.91**	1.63 × 10^−10^	FhaC	Haemolysin activator
FXO12_15125	3311388..3313070		ShlB/FhaC/HecB family hemolysin secretion/activation protein	**−1.28**	1.58 × 10^−7^	FhaC	Haemolysin activator
FXO12_20210 *	Complement (3194188..3195213)		Hemolysin III family protein	0.93	4.70 × 10^−3^	HemH	Heme biosynthetic pathway
FXO12_17340	Complement (3828754..3829305)		RNA polymerase subunit sigma-70	**1.68**	7.65 × 10^−8^	RpoE; sigma70-ECF	Transcriptional control
FXO12_12235	2709169..2709936		Siderophore-interacting protein	**1.41**	3.36 × 10^−2^	SIP	Siderophore interaction
FXO12_07455	1693966..1694364		Low-affinity iron permease family protein	**3.16**	0	Fet4	Inorganic ion (e.g., Fe) iron transport
FXO12_08250	1871305..1872381		Extracellular solute-binding protein	**1.49**	3.54 × 10^−5^	AfuA	Fe^3+^ transport
FXO12_04205	896184..896987		ABC transporter ATP-binding protein	**1.31**	8.75 × 10^−6^	TauB	Nitrate/sulfonate/bicarbonate transport
FXO12_08260	1873401..1874246		ABC transporter permease subunit	**1.07**	1.92 × 10^−4^	COG4132	ABC-type uncharacterized transport system
FXO12_07150	Complement (1625940..1628069)		TonB-dependent siderophore receptor	**−1.12**	1.22 × 10^−4^	PRK10044; CirA	Fe transport
FXO12_06655 *	1476863..1479025		TonB-dependent siderophore receptor	−0.94	2.23 × 10^−3^	PRK10044; CirA	Fe transport
FXO12_24140	Complement (5282386..5283000)		Biliverdin-producing heme oxygenase	**1.58**	7.47 × 10^−7^	HemeO-bac	Heme oxidation in bacteria
FXO12_15500	3405445..3405975		Bacterioferritin	**2.29**	5.67 × 10^−11^	Bfr	Inorganic ion transport and storage
FXO12_11970	2640649..2641119	*bfr*	Bacterioferritin	**−1.44**	2.43 × 10^−7^	Bacterioferritin	Iron binding, transport and storage
FXO12_22240 *	4873628..4874092	*bfr*	Bacterioferritin	−0.88	2.15 × 10^−2^	Bacterioferritin	Iron binding, transport and storage
FXO12_24275	5301834..5303696	*hscA*	Fe-S protein assembly chaperone HscA	**−1.62**	6.30 × 10^−6^	HscA; DnaK; HSP70	Fe-S cluster biogenesis
FXO12_24270 *	5301261..5301782	*hscB*	Co-chaperone HscB	−0.98	5.49 × 10^−3^	HscB	maturation of iron-sulfur cluster-containing proteins
FXO12_24280	5303700..5304041	*fdx*	ISC system 2Fe-2S type ferredoxin	**−1.59**	2.33 × 10^−7^	Fdx_isc	Electron transfer agent
FXO12_11905	2627651..2627974		Ferredoxin family protein	**−1.97**	2.54 × 10^−12^	DUF3470	Electron transfer agent
FXO12_19935	Complement (4427789..4428040)		YfhL family 4Fe-4S dicluster ferredoxin	**−1.21**	2.93 × 10^−4^	di_4Fe-4S_YfhL; PRK07118	Unknwon
FXO12_19255	4281501..4281833	*cyaY*	Iron donor protein Cyay	**−1.18**	9.09 × 10^−5^	CyaY; Frataxin	Iron donor in Fe-S cluster biogenesis
FXO12_23050	5034033..5034557		(2Fe-2S)-binding protein	**1.29**	5.95 × 10^−6^	CoxS	Unknwon

^a–d^, Refer to Table 3 for details on all column constructions. FC of DEGs is in bold. ^e^, retrieved from Conserved Domains Database (CDD) at NCBI (https://www.ncbi.nlm.nih.gov/cdd/) by blasting (BlastP) the corresponding amino acid sequence; ^f^, retrieved from CDD or UniProt database (https://www.uniprot.org/).

## Data Availability

Not applicable.

## Supplementary Materials

The following are available online at https://www.mdpi.com/article/10.3390/microorganisms9040812/s1, Figure S1. The geographical location of sampling site and clinical pathology of wild-caught cunners, Figure S2. Illumina sequencing quality data for the samples (*n* = 6) used in the transcriptomic profiling of *Pseudomonas* sp. J380 under iron-enriched and iron-limited conditions, Figure S3. Growth and hemolysis assays for *Pseudomonas* sp. J380, Figure S4. Gross pathology in cleaner fish (cunners, *Tautogolabrus adspersus,* and lumpfish, *Cyclopterus lumpus*) infected with *Pseudomonas* sp. J380, Figure S5. Evolutionary relationships of taxa based on MLSA, Figure S6. Comparative genomic analyses of *Pseudomonas* sp. J380 and other pseudomonads, Figure S7. Evolutionary relationships of taxa based on whole genomes, Figure S8. Gene structure of the *hut* operon in *Pseudomonas* sp. J380, Figure S9. The *ppqFABCDE* operon involved in pyrroloquinoline quinone biosynthesis in *Pseudomonas* sp. J380, Figure S10. The operons involved in respiratory metabolism in *Pseudomonas* sp. J380, Figure S11. The *alg* operon and its regulators involved in alginate biosynthetic pathway in *Pseudomonas* sp. J380, Figure S12. The *isc* operon in *Pseudomonas* sp. J380. Table S1. Features of the genes utilized for the Multilocus Sequence Analysis (MLSA), Table S2. Comprehensive information of species and their genomes utilized in whole genome comparison and phylogenomics, Table S3. Summary of the RNA-seq reads, Table S4. Comprehensive summary of putative virulence factors identified in *Pseudomonas* sp. J380 genome, Table S5. Comprehensive summary of differentially expressed genes (DEGs) in *Pseudomonas* sp. J380 under iron-limited and iron-enriched conditions as identified by RNA-Seq, Table S6. Comprehensive summary of enriched gene ontology (GO) terms in *Pseudomonas* sp. J380 under iron-limited and iron-enriched conditions, Table S7. Comprehensive summary of enriched KEGG pathways in *Pseudomonas* sp. J380 under iron-limited and iron-enriched conditions, Table S8. Additional genes related to iron homeostasis, File S1. Comprehensive details of GO cluster groups and individual GO terms for iron homeostasis-related DEGs.
