# Supplementary material for: A Novel Marine Pathogen Isolated from Wild Cunners (Tautogolabrus adspersus): Comparative Genomics and Transcriptome Profiling of Pseudomonas sp. Strain J380
Source: Microorganisms. 2021 Apr 12;9(4):812. doi: 10.3390/microorganisms9040812 (PMC8069873; doi:10.3390/microorganisms9040812)
Supplement: Supplementary file 1 [file microorganisms-09-00812-s001.zip › Suppl_Fig_v6.docx]

Article

A Novel Marine Pathogen Isolated from Wild Cunners (*Tautogolabrus adspersus*): Comparative Genomics and Transcriptome Profiling of *Pseudomonas* sp. Strain J380

Navaneethaiyer Umasuthan^∮1^, Katherinne Valderrama^∮1^, Ignacio Vasquez ^1^, Ahmed Hossain ^1^, Trung Cao ^1^, Hajarooba Gnanagobal ^1^, Cristopher Segovia ^1^, Jennifer Monk ^2^, Danny Boyce ^2^, and Javier Santander ^1^*

^1^Marine Microbial Pathogenesis and Vaccinology Laboratory, Department of Ocean Sciences, Memorial University of Newfoundland, Canada; [navaumasuthan@gmail.com](mailto:navaumasuthan@gmail.com) (N.U.); [kvalderrama@mun.ca](mailto:kvalderrama@mun.ca) (K.V.); [ivasquezsoli@mun.ca](mailto:ivasquezsoli@mun.ca) (I.V.); [ahossain@mun.ca](mailto:ahossain@mun.ca) (A.H.); [ttcao@mun.ca](mailto:ttcao@mun.ca) (T.C.); [hgnanagobal@mun.ca](mailto:hgnanagobal@mun.ca) (H.G.); [cwsegovia@mun.ca](mailto:cwsegovia@mun.ca) (C.S.)

^2^Dr. Joe Brown Aquatic Research Building (JBARB), Department of Ocean Sciences, Memorial University of Newfoundland, Logy Bay, NL A1C 5S7, Canada; [jmonk@mun.ca](mailto:jmonk@mun.ca) (J.M.); [dboyce@mun.ca](mailto:dboyce@mun.ca) (D.B.)

^∮^ These authors contributed equally

***** Correspondence: jsantander@mun.ca; Tel.: +1(709)8643268

**Supplementary**

**Figures**


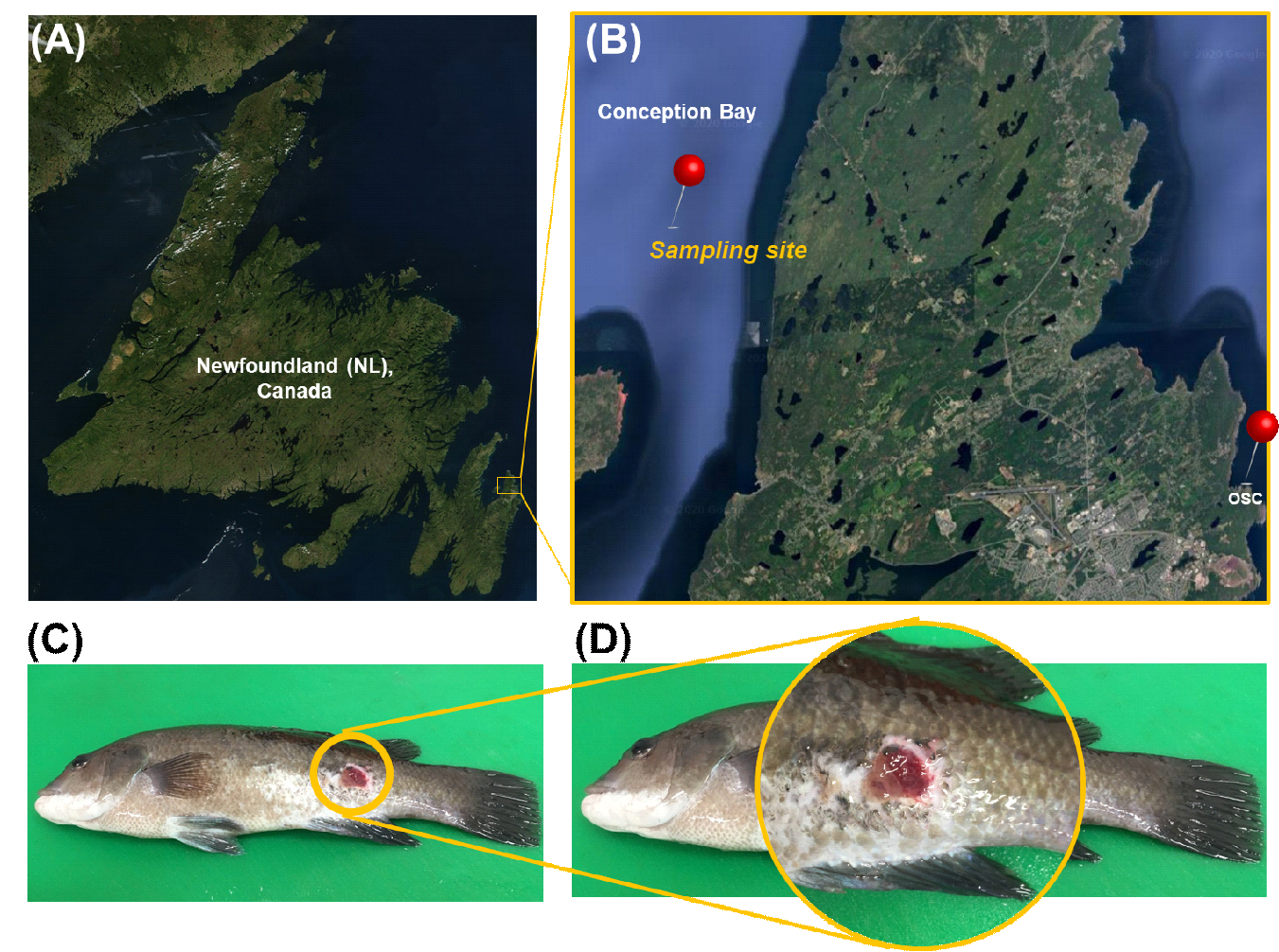


**Figure S1.** The geographical location of sampling site and clinical pathology of wild-caught cunners. (**A, B**) The sampling region was located in Newfoundland seashore at Conception Bay, and sampling was conducted in spring, 2017. OSC, Ocean Sciences Centre; Image source: Google Earth. (**C**) Wild-caught cunner with characteristic skin ulcers. (**D**) Magnified view of the wound caused by a previously uncharacterized infection. Clinical signs included scale-loss, hemorrhage, skin damage, and ulcers. The internal organs (i.e., spleen, liver, and head kidney) of the infected cunner were harvested, homogenized, and plated on TSA. Developed colonies represented the disease-causing bacterium, *Pseudomonas* sp. strain J380, which was then characterized in detail in the current study.


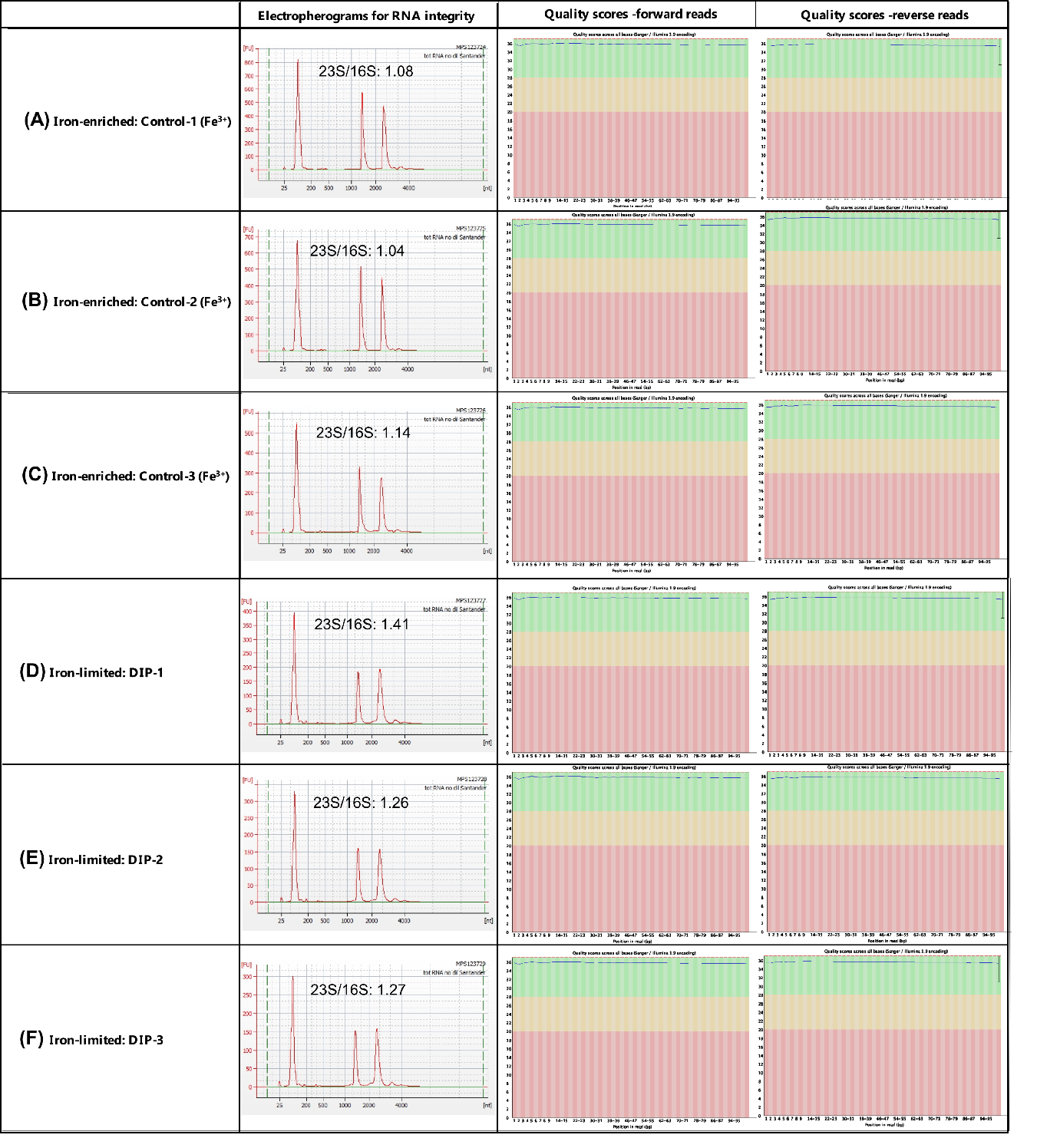


**Figure S2**. Illumina sequencing quality data for the samples (*n* = 6) used in the transcriptomic profiling of *Pseudomonas* sp. J380 under iron-enriched (**A**-**C**) and iron-limited conditions (**D**-**F**). The 23S/16S ratios of RNA samples are indicated in the electropherograms. The x-axis of quality score (QS) plots is ‘position in read (bp)’. QS across all bases for forward (Read-1) and reverse (Read-2) raw reads from the Fast QC report showed that the reads are virtually perfect in quality, having zero errors and ambiguities.


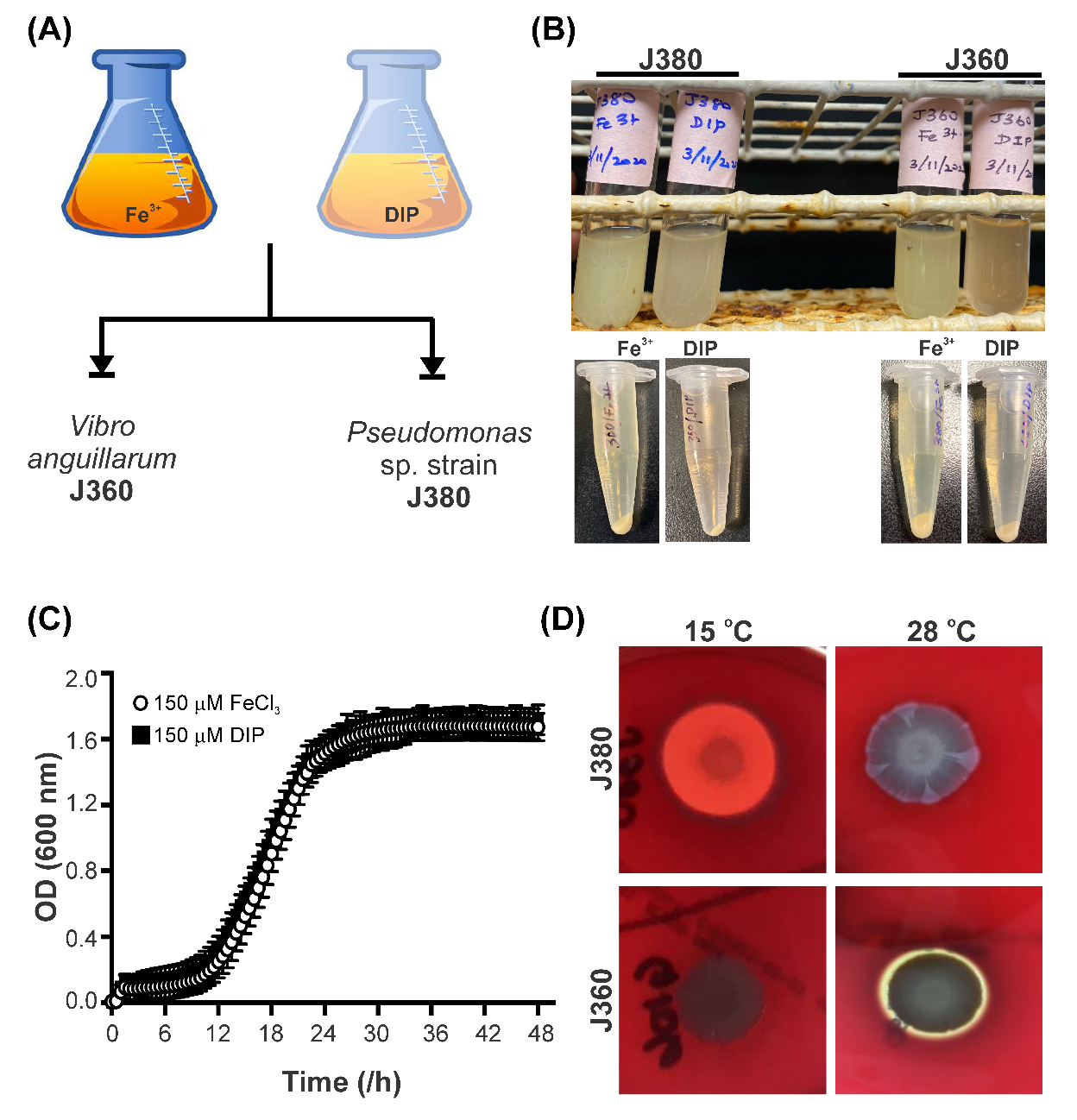


**Figure S3.** Growth and hemolysis assays for *Pseudomonas* sp. J380. **(A)** Brief experimental design for growth assay under iron-enriched (Fe^3+^-supplemented) and iron-limited (DIP-supplemented) conditions for *Vibrio anguillarum* J360 (control) and *Pseudomonas* sp. J380. **(B)** Cultures for each strain were grown for 24 h (upper panel) and harvested (lower panel). (**C**) Quantitative growth kinetics of *Pseudomonas* sp. J380 under iron-enriched and iron-limited conditions. **(D)** Hemolysis assay was conducted using blood sheep agar plates with *V. anguillarum* J360 and *Pseudomonas* sp. J380 at 15 °C and 28 °C. Plates were pictured after 48 h incubation at indicated temperatures. (Image source for flask (**A**): <https://www.cleanpng.com/>).


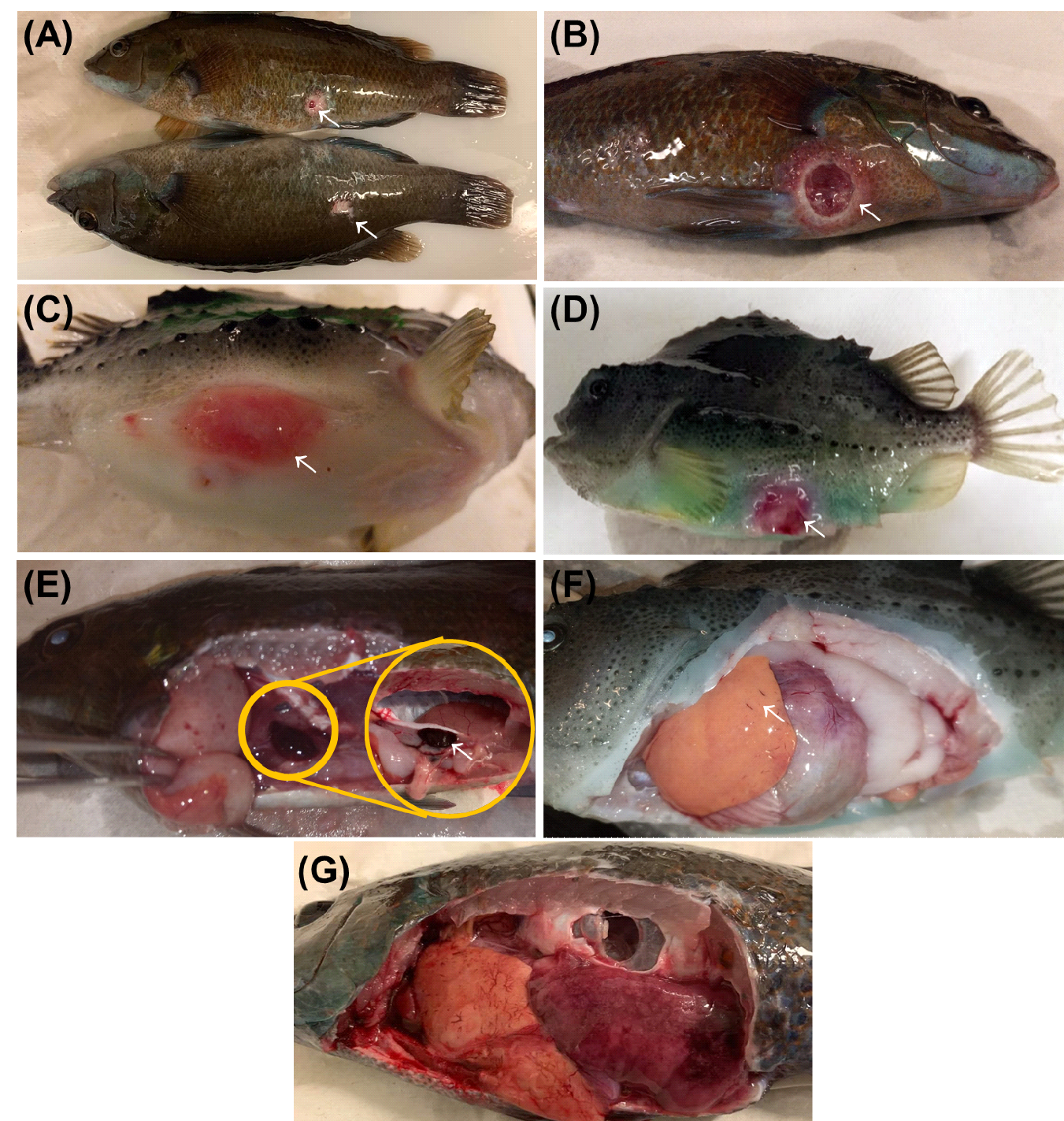


**Figure S4.** Gross pathology in cleaner fish (cunners, *Tautogolabrus adspersus,* and lumpfish, *Cyclopterus lumpus*) infected with *Pseudomonas* sp. J380. The white arrow indicates the spot with a specific clinical sign. **(A-D)** External clinical signs. **(E, F)** Abdominal dropsy revealing internal symptoms. Skin ulceration with hemorrhagic borders at early **(A, C)** and advanced (i.e., chronic; **B, D)** stages in cunners and lumpfish, respectively. For instance, splenomegaly in cunner **(E)** and hemorrhagic petechiae in liver of lumpfish at the initial stage **(F)** and cunner in advanced stage (**G**) are shown. **(C)** At 13 dpi and **(D)** At 20 dpi.


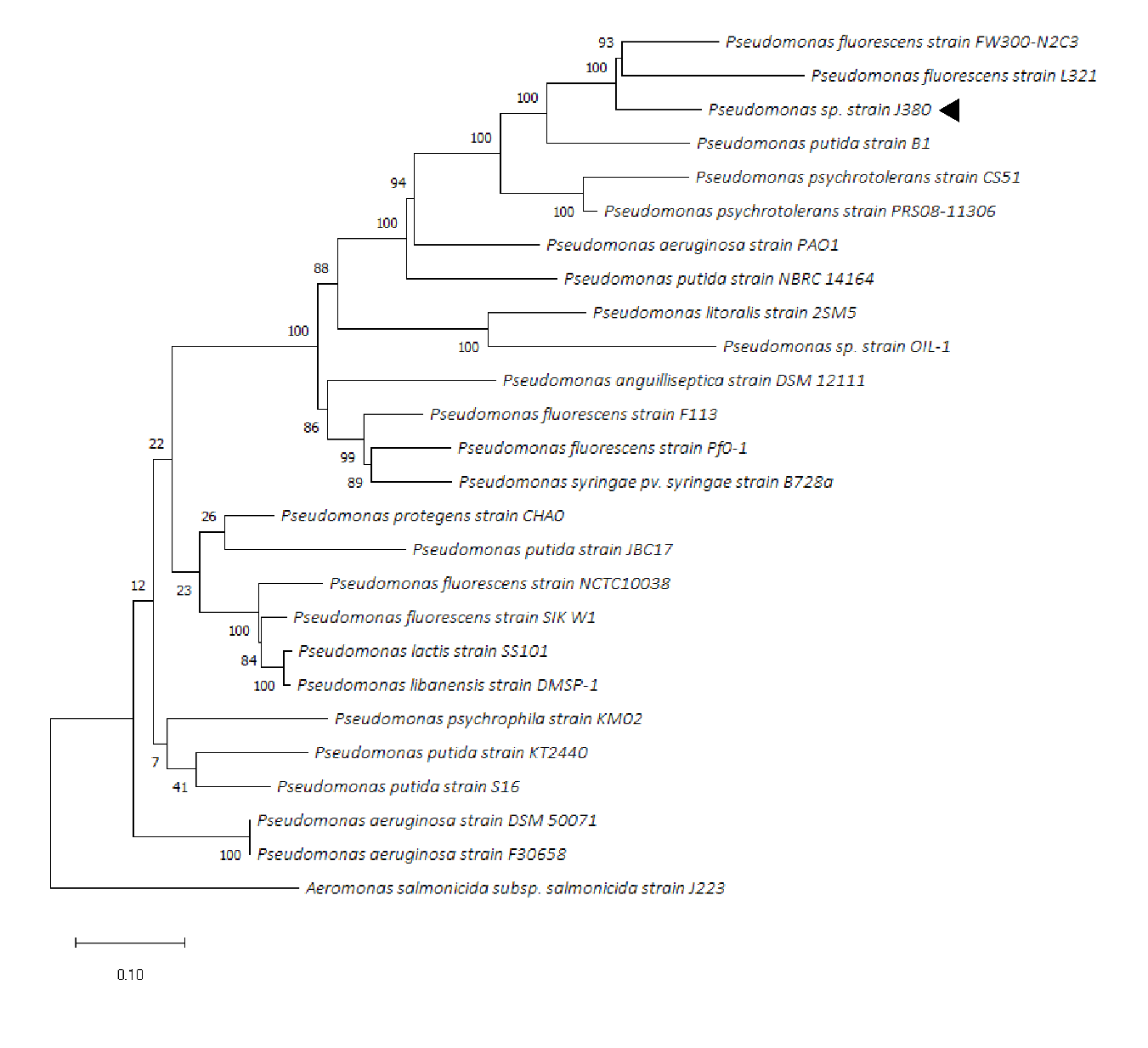


**Figure S5.** Evolutionary relationships of taxa based on MLSA. The evolutionary history was inferred using the Neighbor-Joining method. The optimal tree with the sum of branch length = 3.43064748 is shown. The tree is drawn to scale, with branch lengths in the same units as those of the evolutionary distances used to infer the phylogenetic tree. The evolutionary distances were computed using the Jukes-Cantor method and are in the units of the number of base substitutions per site. This analysis involved 26 nucleotide sequences. Codon positions included were 1st+2nd+3rd+Noncoding. All ambiguous positions were removed for each sequence pair (pairwise deletion option). There were a total of 12770 positions in the final dataset. Evolutionary analyses were conducted in MEGA X.


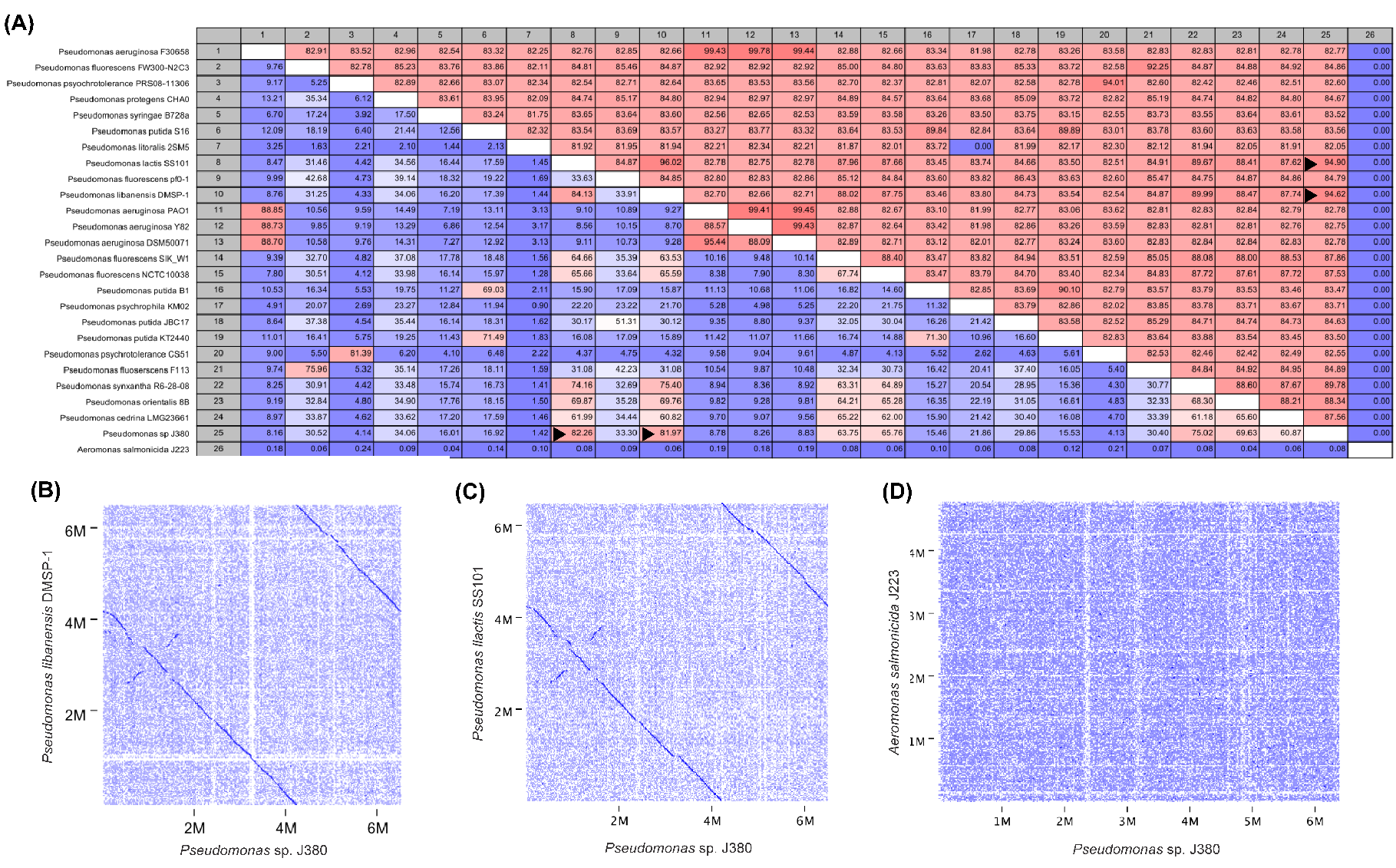


**Figure S6.** Comparative genomic analyses of *Pseudomonas* sp. J380 and other pseudomonads. **(A)** Average nucleotide identity (ANI) comparison matrix for *Pseudomonas* sp. J380 whole genome with selected bacterial genomes from *Pseudomonas* genus. Identity percentage parameters for annotated genes were set up as minimum similarity of 0.8 and minimum length 0.8. Analyses were conducted in CLC Genomic Workbench v20 (CLC Bio, Qiagen). Higher identity values of *P. libanensis* DMSP-1 and *P. lactis* SS101 with *Pseudomonas* sp. J380 genome have been red-boxed. Upper triangle region, ANI; lower triangle region, AP (average percent identity). **(B-D)** Whole-genome dot plots for the comparison of *Pseudomonas* sp. J380 genome with selected genomes sharing highest identities (B, *P. lactis* SS101 and C, *P. libanensis* DMSP-1 ) and out-group (D, *A. salmonicida* J223)**.** Each dot represents the ‘ortholog’ relationship of genes in two species being compared. Blue lines in B and C indicate the identical genes in the two species being compared, which are in reverse orientation.


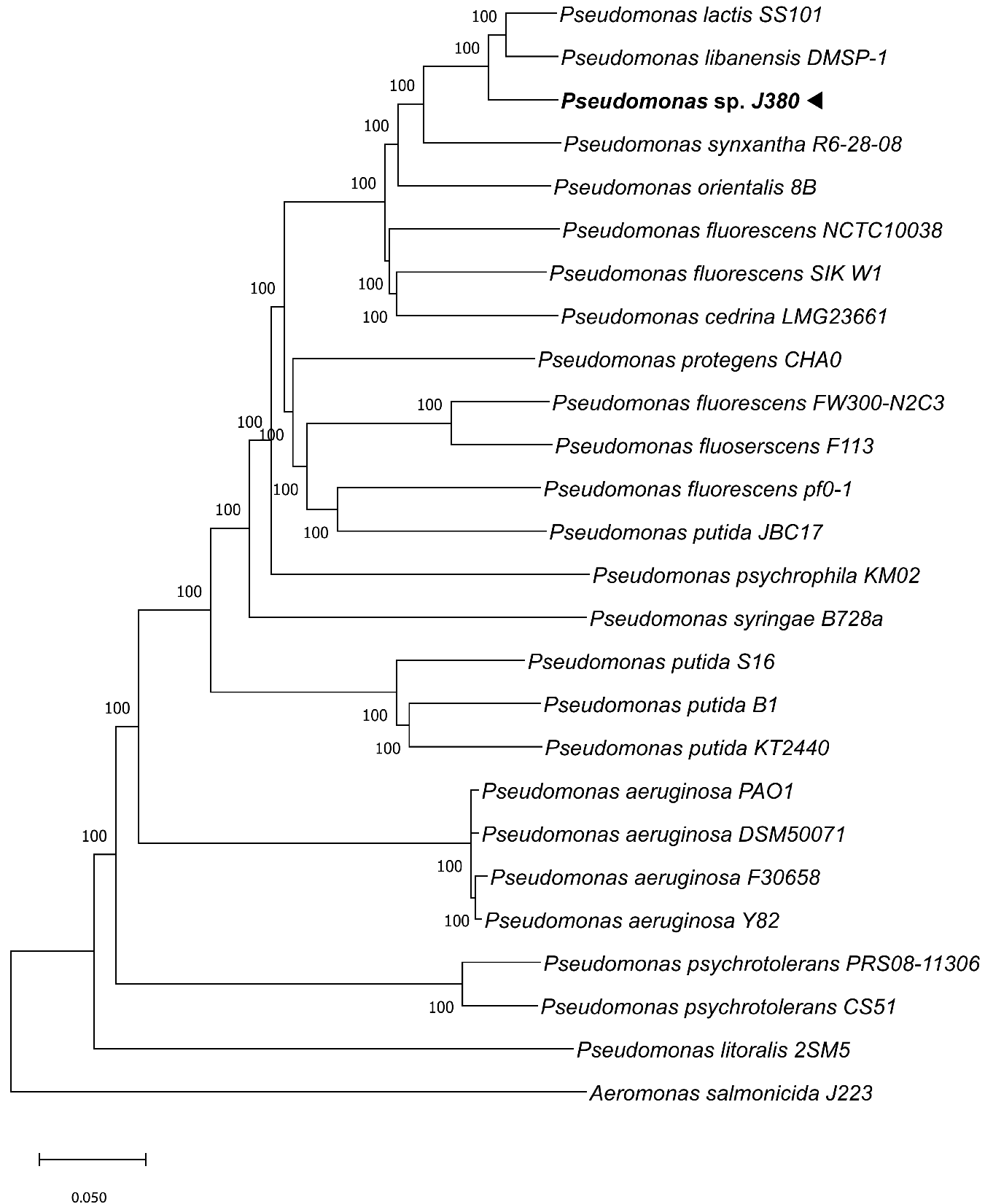


**Figure S7**. Evolutionary relationships of taxa based on whole genomes**.** The evolutionary history was inferred using the Neighbor-Joining method. The tree is drawn to scale, with branch lengths in the same units as those of the evolutionary distances used to infer the phylogenetic tree. The evolutionary distances were computed using the Jukes-Cantor method and are in the units of the number of base substitutions per site. All ambiguous positions were removed for each sequence pair (pairwise deletion option). Evolutionary analyses were conducted in MEGA X. *Aeromonas salmonicida* J223 was used as an outgroup.


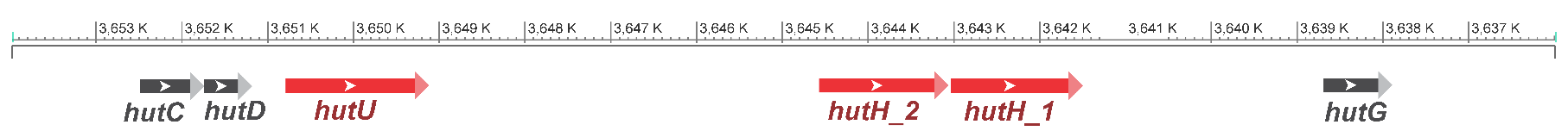


**Figure S8.** Gene structure of the *hut* operon in *Pseudomonas* sp. J380. Genomic coordinates are shown above the genes. *Pseudomonas* sp. J380 *hut* operon consists of 5 genes, including 2 copies of *hutH* and a single copy of each *hutC*, *hutD*, *hutU* and *hutG*. The red arrows indicate the genes that are up-regulated under iron-limited conditions. Black arrows indicate the genes with no significant change in transcript expression under iron-limited conditions. Refer to Table 3 for complete information about the genes of *hut* operon.


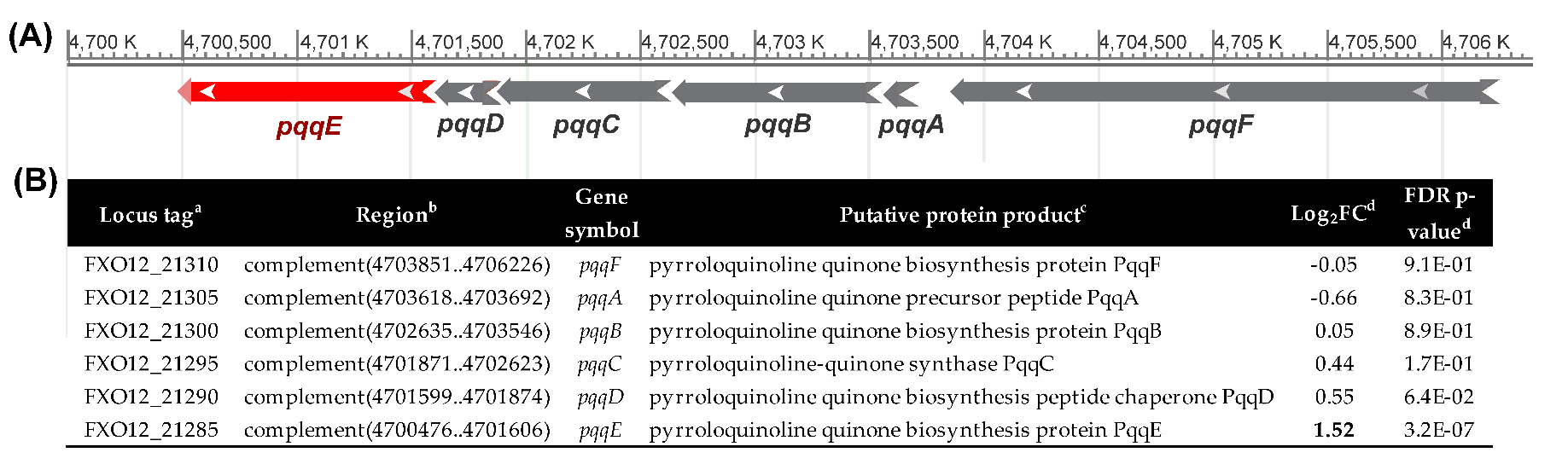


**Figure S9.** The *ppqFABCDE* operon involved in pyrroloquinoline quinone biosynthesis in *Pseudomonas* sp. J380. (**A**) Gene structure of the *ppqFABCDE* operon. Genomic coordinates are shown above the genes. *Pseudomonas* sp. J380 *ppqFABCDE* operon consists of 6 genes. The red arrows indicate the up-regulated gene(s) under iron-limited conditions. Black arrows indicate the genes with no significant change in transcript expression under iron-limited conditions. (**B**) Comprehensive details of genes in *ppqFABCDE* operon. a, gene-specific identifier in NCBI genome database; b, spanning of the coding sequence; c, obtained from UniProt, NCBI, or *Pseudomonas* Genome DB (PGDB; https://pseudomonas.com/); d, data from RNA-Seq. FC of DEGs is in bold.


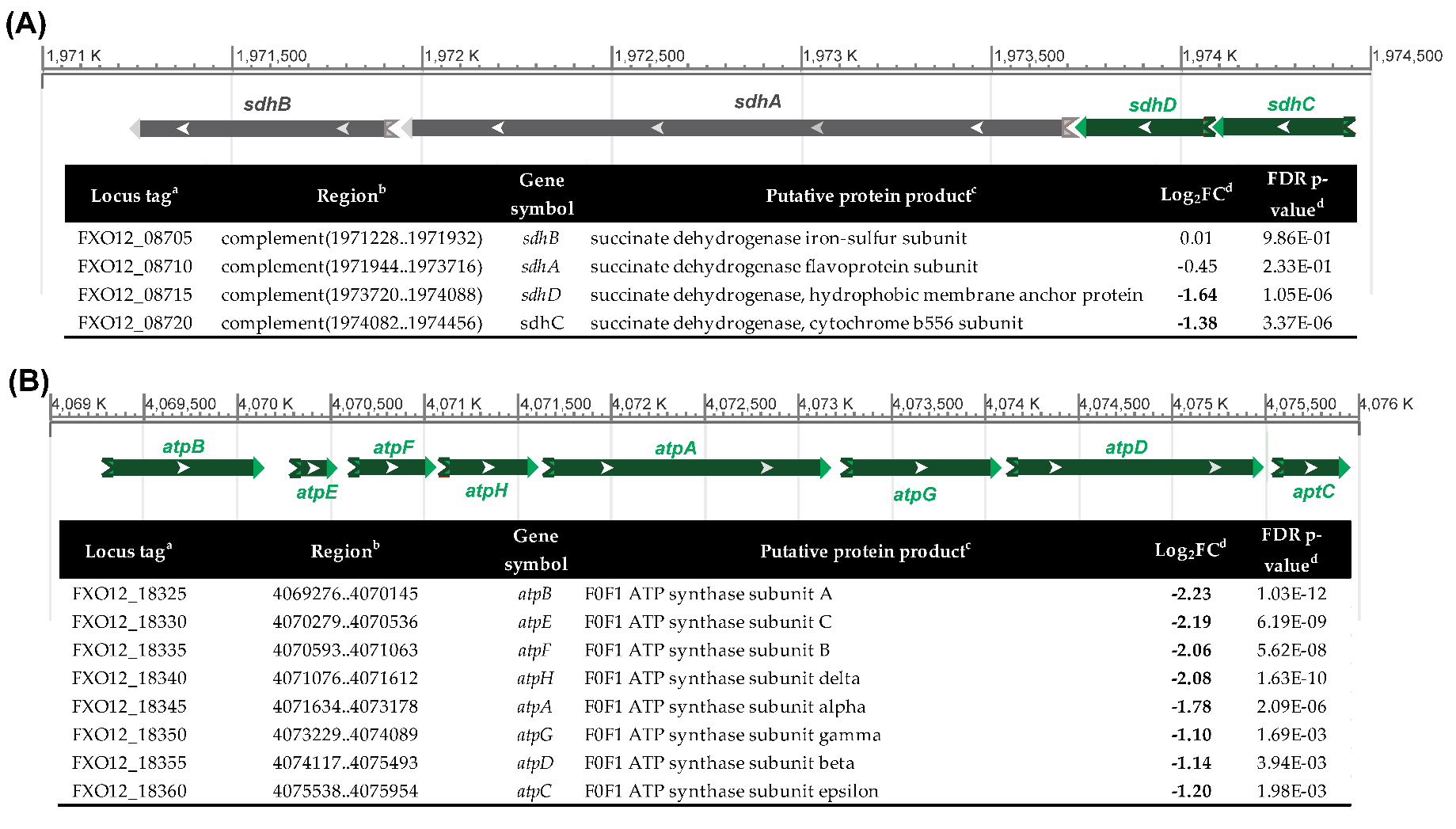


**Figure S10.** The operons involved in respiratory metabolism in *Pseudomonas* sp. J380. (A) Gene structure of the *sdhCDAB* cluster. Genomic coordinates are shown above the genes. *Pseudomonas* sp. J380 *sdhCDAB* operon consists of 4 genes. (B) Gene structure of the *atp* operon. The green arrows indicate the down-regulated gene(s) under iron-limited conditions. Black arrows indicate the genes with no significant change in transcript expression under iron-limited conditions. Comprehensive details of genes in operons are presented below in inset tables for each operon. a, gene-specific identifier in NCBI genome database; b, spanning of the coding sequence; c, obtained from UniProt, NCBI, or *Pseudomonas* Genome DB (PGDB; https://pseudomonas.com/); d, data from RNA-Seq. FC of DEGs is in bold.


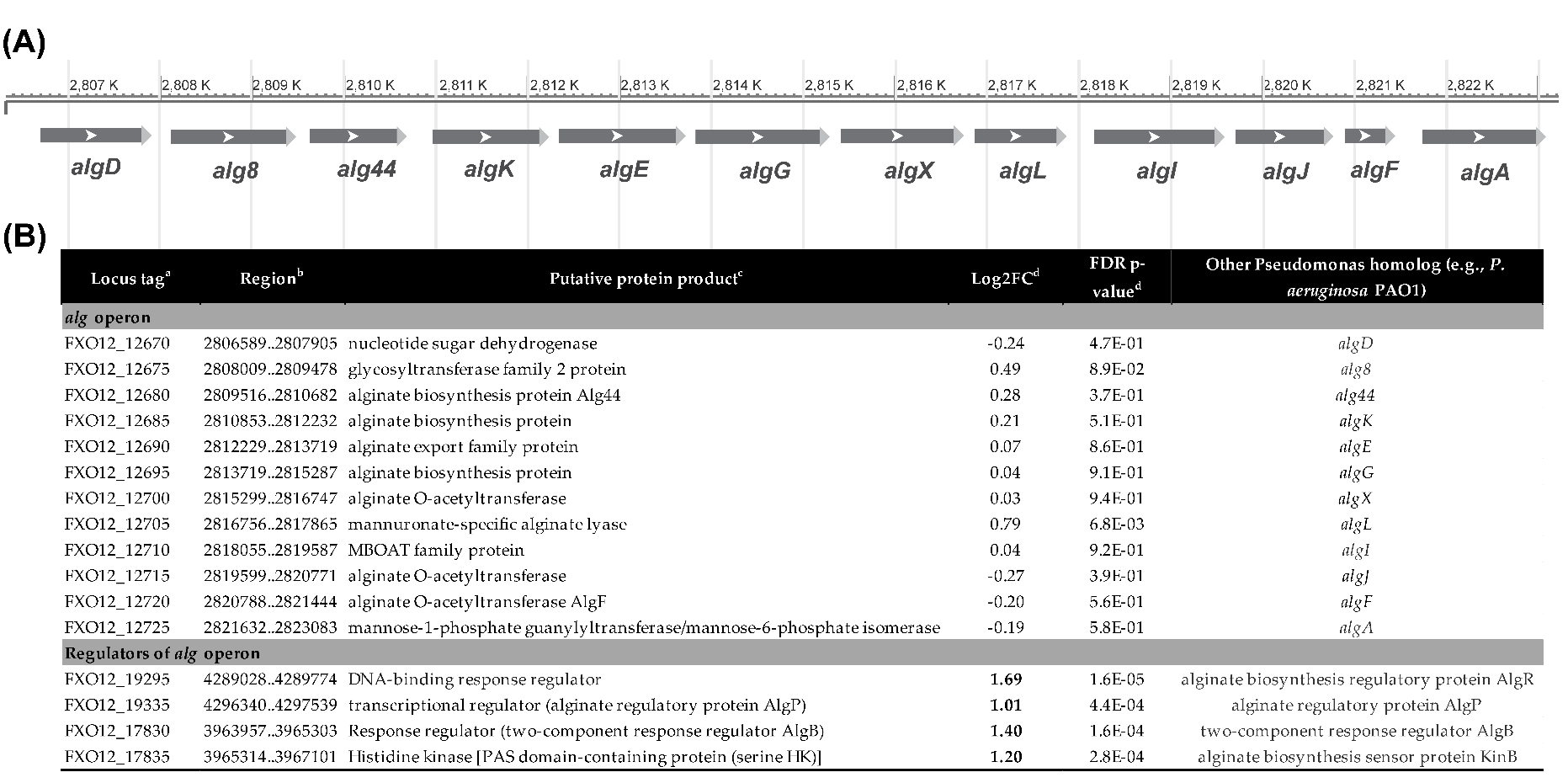


**Figure S11.** The *alg* operon and its regulators involved in alginate biosynthetic pathway in *Pseudomonas* sp. J380. (A) Gene structure of the *alg* operon. Genomic coordinates are shown above the genes. *Pseudomonas* sp. J380 *alg* operon is consisted of 12 genes. None of them were DEGs in the current study. Black arrows indicate the genes with no significant change in transcript expression under iron-limited conditions. (B) Comprehensive details of genes in *alg* operon and its regulators. a, gene-specific identifier in NCBI genome database; b, spanning of the coding sequence; c, obtained from UniProt, NCBI, or *Pseudomonas* Genome DB (PGDB; https://pseudomonas.com/); d, data from RNA-Seq.


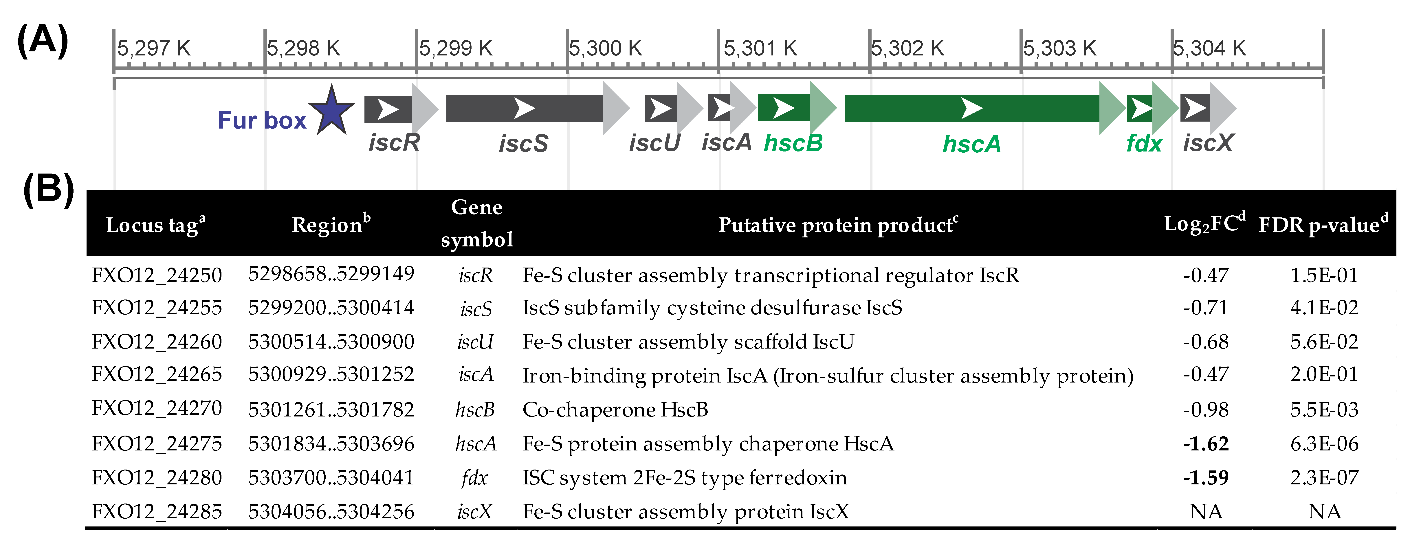


**Figure S12.** The *isc* operon in *Pseudomonas* sp. J380. (A) Gene structure of the *isc* operon. Genomic coordinates are shown above the genes. *Pseudomonas* sp. J380 *isc* operon consists of 8 genes in the order of *iscRSUABA-fdx-iscX*. The green arrows indicate the genes that are down-regulated under iron-limited conditions. *hscB* was absent in DEG list (log_2_FC=-0.98). Black arrows indicate the genes with no significant change in transcript expression under iron-limited conditions. A putative Fur box (star; score 7.61) was predicted by Virtual Footprint (<http://www.prodoric.de/vfp/vfp_promoter.php>). (B) Comprehensive details of genes in *isc* operon. a, gene-specific identifier in NCBI genome database; b, spanning of the coding sequence; c, obtained from UniProt, NCBI, or *Pseudomonas* Genome DB (PGDB; https://pseudomonas.com/); d, data from RNA-Seq. FC of DEGs is in bold.
